# A Biomechanical Analysis of Ventral Furrow Formation in the Drosophila Melanogaster Embryo

**DOI:** 10.1371/journal.pone.0034473

**Published:** 2012-04-12

**Authors:** Vito Conte, Florian Ulrich, Buzz Baum, Jose Muñoz, Jim Veldhuis, Wayne Brodland, Mark Miodownik

**Affiliations:** 1 Institute for Bioengineering of Catalonia, Barcelona, Spain; 2 Skirball Institute of Biomolecular Medicine, New York Hospital, New York, New York, United States of America; 3 Medical Research Council Laboratory for Molecular Cell Biology, University College London, London, United Kingdom; 4 Laboratori de Càlcul Numèric, Department of Applied Mathematics III, Universitat Politècnica de Catalunya, Barcelona, Spain; 5 Department of Civil and Environmental Engineering, University of Waterloo, Waterloo, Canada; 6 Department of Biology, University of Waterloo, Waterloo, Canada; 7 Mechanical Engineering Department, University College London, London, United Kingdom; University of North Carolina at Chapel Hill, United States of America

## Abstract

The article provides a biomechanical analysis of ventral furrow formation in the *Drosophila* melanogaster embryo. Ventral furrow formation is the first large-scale morphogenetic movement in the fly embryo. It involves deformation of a uniform cellular monolayer formed following cellularisation, and has therefore long been used as a simple system in which to explore the role of mechanics in force generation. Here we use a quantitative framework to carry out a systematic perturbation analysis to determine the role of each of the active forces observed. The analysis confirms that ventral furrow invagination arises from a combination of apical constriction and apical–basal shortening forces in the mesoderm, together with a combination of ectodermal forces. We show that the mesodermal forces are crucial for invagination: the loss of apical constriction leads to a loss of the furrow, while the mesodermal radial shortening forces are the primary cause of the internalisation of the future mesoderm as the furrow rises. Ectodermal forces play a minor but significant role in furrow formation: without ectodermal forces the furrow is slower to form, does not close properly and has an aberrant morphology. Nevertheless, despite changes in the active mesodermal and ectodermal forces lead to changes in the timing and extent of furrow, invagination is eventually achieved in most cases, implying that the system is robust to perturbation and therefore over-determined.

## Introduction

**Figure 1:**
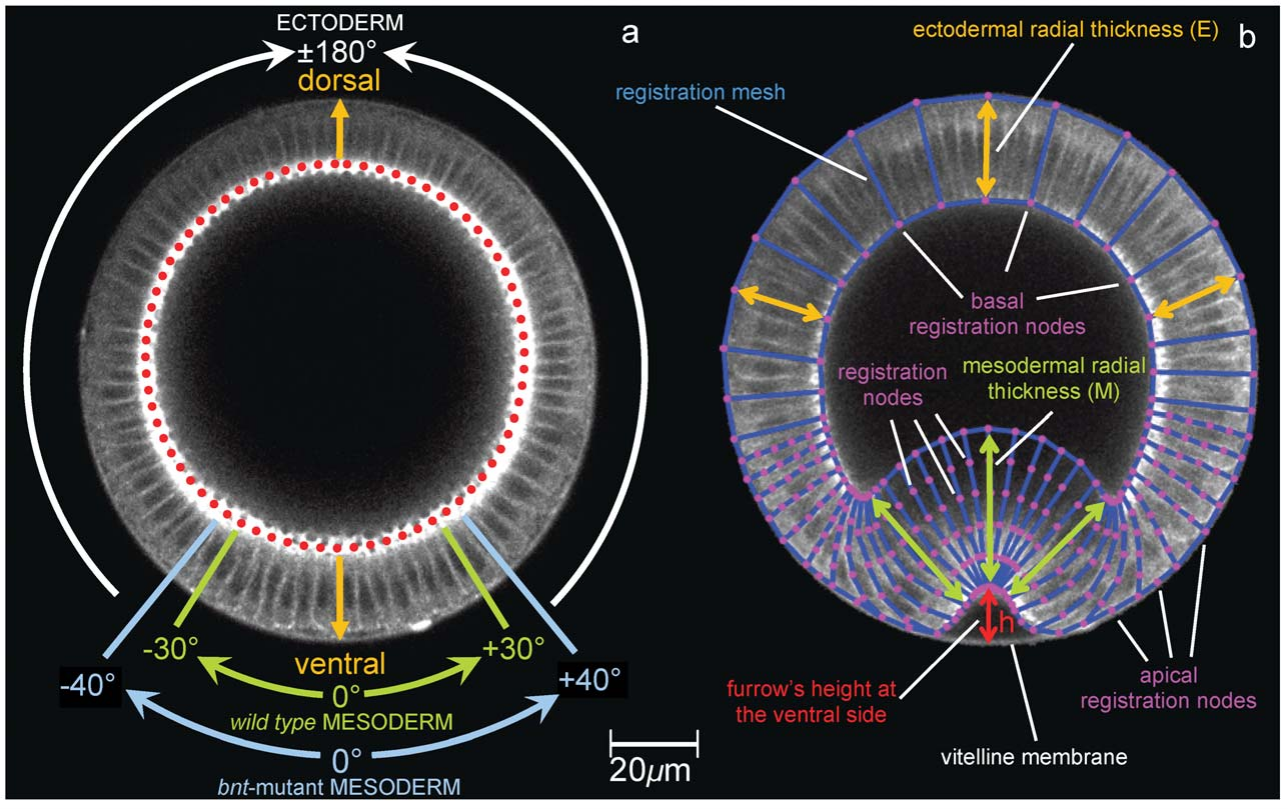
Digitisation procedure and epithelial fate. (**a**) The angular coordinate system is defined in the reference configuration at t = −10.43 min, which is considered as initial configuration. The presumptive mesoderm is defined as the tissue that ultimately forms the ventral furrow in the *wt* embryo. By tracking backward in time, this tissue was found to be nominally 18 cells wide in the *wt* embryo and to span a ±44° angle about the dorsal (D)-ventral (V) midline at the reference instant t = −10.43 min. Only a mesodermal sub-region is thought to be active by undergoing apical constriction. Myosin II that is initially localized on the basal side of the epithelium (red-dotted line) vanishes in this sub-region to reappear apically (on the opposite end of cells). In the wild type and *bnt*-mutant embryos, this sub-region was respectively found to span approximately ±30° (green lines) and ±40° (blue lines) astride the dorsal-ventral midline. (**b**) Digitisation procedure of an intermediate-stage frame from the *in-vivo* imaged sequence of a *wt* embryo: a polygonal mesh (blue lines) has been overlaid on the tissue to track displacements at nodal points, referred to as registration nodes (magenta dots). Polygonal partitions can correspond to single cells, multiple cells or sub-cellular regions, depending on the measurements refinement required in that particular portion of tissue. Arrows point the radial thickness of the epithelium, which was measured on the mesh as the distance between basal and apical node along the same side of a given cell. The height of the furrow (h) has been measured as that of the most ventral nodal point with respect to its position in the reference frame at t = −10.43 min.

Ventral furrow invagination in *Drosophila melanogaster* provides a model system uniquely suited for investigating the cellular and molecular mechanisms of morphogenesis [1], [2]. The system possesses three important advantages: its relatively simple morphology, a diversity of cell behaviours and the potential for genetic analyses. The process begins as soon as cellularisation is complete and the embryo is composed of a relatively uniform single layer of columnar epithelial cells, surrounded by a shell composed of a vitelline membrane which contains a liquid yolk. This organisation can be readily appreciated in cross–section, where the epithelial blastoderm forms a circular array of columnar cells with their apical–basal axes aligned radially, and their apical surfaces facing outwards, see Fig. 1a. During invagination, over a period of approximately 20 minutes, a single morphogenetic movement transforms this geometry into a multi-layered structure by inducing the internalisation of cells most ventrally–positioned in the embryonic epithelium, see Fig. 1b.

The site at which the ventral furrow is formed is determined by two ventrally–expressed transcription factors, Twist and Snail [2], [3]. A combination of local *twist*–induced expression of mesodermal genes and *snail*–mediated repression of ectodermal cell fate appears to bring about ventral furrow invagination, via cell biological events such as apical flattening, apical constriction, apical–basal lengthening, apical–basal shortening and basal expansion.

Ventral furrow formation is thought to be driven by coordinated forces induced by individual cells which generate changes in the whole tissue [4], [5], [6]. The first observable event in this process is the flattening of the apical surfaces of cells within the most ventral region of the cellular blastoderm [6], [7]. This is followed by the constriction of the apical domain of scattered cells within this population. As these apical constriction events become more widespread they cause the formation of a shallow indentation along the ventral surface of the embryo [8]. At the same time, cells within the furrow lengthen along their apical–basal axis, reaching up to 1.7 times their original height [4]. Once the furrow has formed, its constituent cells then begin to shorten back to their original length, whilst keeping their apical ends constricted [4], resulting in a wedge–like form. This second change in cell shape has been proposed to constitute the final force driving furrow internalisation [4], [6]. Additionally, however, it has been suggested by a number of authors that the lateral and dorsal ectodermal cells could help to drive ventral furrow invagination by pushing laterally on the sides of the prospective mesoderm; facilitating inward buckling and reinforcing internalisation of the ventral furrow [4], [6], [9], [10].

There has been much previous work to model the mechanics of invagination in various organisms such as the pioneering work of Odel et al. [8], and the work by Davidson et al. [11] on the invagination of sea urchin; as well as more recent studies by Pouille and Farge on *Drosophila melanogaster* [12], by Chen and Brodland (2009) on *Xenopus* [13], by Sherrard et al. on *Ascidian* [14], by Allena et al. on *Drosophila melanogaster* [15], and by Tamulonis et al. (2011) on *Nematostella vectensis* [16]. These studies show that there are various ways in which invagination can be achieved in different organisms and they confirm that a combination of modelling and experiment are usually required to identify the active forces involved.

Recently we introduced a new technique called video force microscopy (VFM) to determine the active forces in the embryo during ventral furrow invagination on *Drosophila melanogaster* from time-lapse images [1]. The method assumes that the forces at work in an embryo can be decomposed into active and passive contributions, where active forces act along cell boundaries and originate from subsystems that do mechanical work to the system (e.g. actino–myosin contraction) and passive forces dissipate energy (e.g. viscous cytoplasm). This analysis revealed that ventral furrow formation is driven by a combination of apical constriction and apical–basal shortening forces in the mesoderm as well as a combination of ectodermal basal constriction (i.e. constriction of the basal apices of ectodermal cells) and ectodermal apical–basal shortening forces. Importantly the forces in the mesoderm are found to vary in an approximately parabolic fashion with time and angular position, while forces along the basal surface of the ectoderm are found to vary less in time and to be nearly uniform with position.

VFM analysis makes use of a 2D *in silico* model of the embryo's cross section and is based on a geometry whose material properties are assigned and whose form is shaped in order to exactly mirror the *in vivo* cross–section of the real embryo at an initial reference time. It takes as input the embryo deformations observed *in vivo* and outputs an approximate set of forces necessary to produce them [1]. We will here refer to this model as the *inverse* model because it allows *in silico* computation of embryonic tissue dynamics (cause) once both its material properties and kinematics (effects) are assumed to be known.

**Figure 2:**
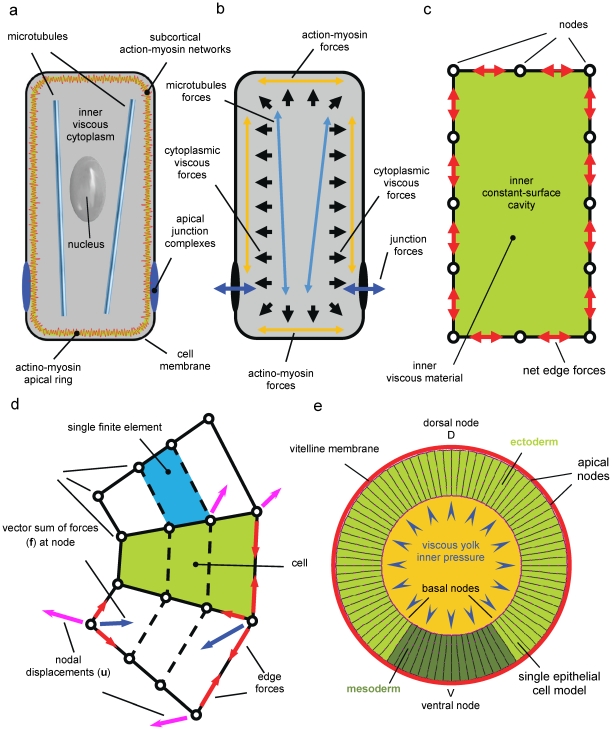
Schematic of the multi-scale model of the 2Dsection of the *Drosophila* embryo. (**a**) Biological architecture of the *Drosophila* single epithelial cell. (**b**) Each architectural constituent of the epithelial cell plays a structural role during cell shape changes. Such roles can be either active or passive, depending on whether that constituent is able to generate force or it is deformed by forces actively generated by neighbouring elements. Actin-myosin forces are known to be active systems for force generation whereas microtubules are known to maintain stiffness. Apical junction complexes, instead, keep cell junctions connected and, therefore, transmit forces from one cell to other cells in the epithelium. The inner cytoplasm is a viscous incompressible fluid that resists and responds to cell compression by exerting an inner pressure. (**c**) In order to build a finite element model of the single epithelial cell, the phenotypical effects of the complex force fields originating from the molecular level can be thought as an equivalent field of net forces acting along the cell edges. The incompressibility of the inner cytoplasm can be simulated by enforcing the surface of the region enclosed by the cell membrane to remain constant in time. (**d**) Individual cells (green) are broken into quadrilateral finite elements (blue), which are used to calculate the forces generated by their passive (viscous) components during each time step t*_i_* of the simulation. Elements are joined to each other at nodes, and the set of forces (blue arrows) that would be needed to drive a particular set of nodal motions **u**_i_ (magenta arrows) is denoted by **f**_i_. These passive forces **f**_i_ and their corresponding displacements **u**_i_ are related by Eqn. (1). The forces generated by active cellular components are resolved into edge forces which act on both of the nodes that mark their ends. Some of these edge forces are shown (red arrows), as is their vector sum at a representative node (blue arrows). Similar vector sums for each node give rise to the collection of nodal driving forces **f**_i_* which are set to be equal to the passive forces **f**_i_ generated by the cytoplasm. Setting these forces equal to each other means that the vectorial driving forces acting at each node are just balanced by viscous resistance from the cytoplasm at that node. (**e**) A finite element model of the 2D section of the Drosophila embryo is built by matching to *in vivo* images at the cellular level. 2D geometry has been sized on the *in vivo* dimensions of the wild type embryo at the reference initial instant t = −10.43 min (fig. 1b–2a). Mesodermal region covers approximately 60 degrees across the ventral point V and is highlighted in dark green, whereas ectodermal region is highlighted in bright green. The presence of the vitelline membrane has been simulated by constraining the motion of apical nodes unilaterally, in such a way that apical nodes could not displace at distances from the centre of the circular epithelium greater than the radius of the circular vitelline membrane (red circle). The effect of the presence of the inner viscous yolk in the real embryo has been modelled, instead, by imposing a user defined pressure to the basal nodes. The value of the pressure is than chosen in such a way that surface variations of the inner yolk region (orange area) are approximately of the same magnitude of *in vivo* yolk area variations (Fig. S1).

In the present work we go beyond VFM analysis by presenting a study of the role of each of the driving forces previously measured as well as by investigating the importance of their relative timing and their possible redundancy in the system. This study moves from an initial validation of the VFM technique showing that deformations observed *in vivo* are obtained again when forces measured through VFM are assigned to the material geometry of the inverse model. In order to better classify and discriminate the actual forces acting in the model we adopt a material geometry that is simplified in shape with respect to the one used by the inverse model, while still sharing the same material properties (Fig. 2e). Also, in order to facilitate the articulation of the study we present here, we did not use the complete *in vivo* force data set collected through VFM. Instead, we use an important approximation to discretise forces acting at each time step. This allows us to compute the morphological changes that each individual force drives by performing a systematic and quantitative perturbation analysis of the system by turning forces on and off and changing their relative timing.

The resulting approach detailed in this paper constitutes a new 2D *in silico* model (Fig. 2), which will be referred to as the *forward model* as opposed to the 2D *in silico* inverse model utilized in the VFM analysis. We show this forward model does yield a quantitatively accurate ventral furrow formation when using our measured forces. We do this by presenting in unprecedented fine details quantification of morphogenetic kinematics and cellular shapes changes during ventral furrow invagination in Drosophila wild type (*wt*) embryos as well as *armadillo* (*arm*), *concertina t*48 (*cta/t48*) and *bicoid, nanos, torso–like* (*bnt*) mutant embryos.

Our experimental and simulation perturbation analyses reveals that ectodermal forces play a minor role in furrow formation: without ectodermal forces the furrow does not close properly and forms a hole in its centre. It also shows that although perturbation of the mesodermal and ectodermal driving forces affects the height of the furrow and thickness of the mesoderm, the process of invagination is surprisingly robust.

## Results

### A combination of active forces is required to drive ventral furrow invagination

**Figure 3:**
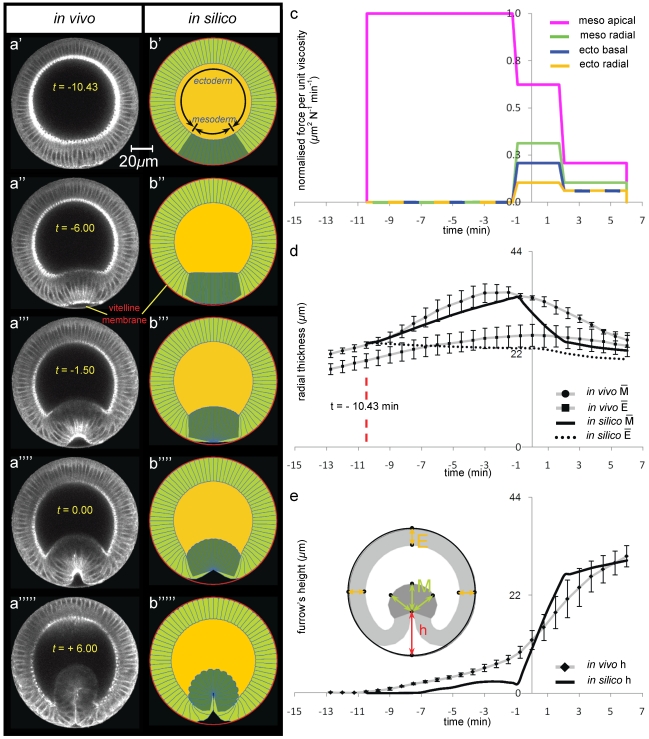
Modelling ventral furrow invagination: wild type genotype. (**a**) Selected multi-photon images (columns) of *in vivo* transverse cross-sections of a wild type embryo during ventral furrow formation. The embryo is labelled with Sqh-GFP and is oriented with its dorsal surface upward (Fig. 1a). The time interval between successive frames is 45 sec. Time zero was set by using apical-basal cell height profiles on the dorsal side (Fig. S2a). (**b**) Selected frames (columns) of *in silico* transverse cross-sections of a wild type genotype. The embryo's 2D geometry is oriented with its dorsal side upward. Undeformed initial configuration (panel b′) corresponds to *in vivo* deformations at t = −10.43 min (see panel d). (**c**) *In silico* force distributions utilised to simulate invagination in b). Total invagination interval [−1.43, 6 min] has been subdivided in the three subintervals [−10.43 min, 1.2 min], [1.2 min,2 min] and [2 min, 6 min] respectively referred to as the *first*, *second* and *third invagination intervals*. Force trends are a discretisation of the *in vivo* force distribution available in Brodland et al. [1]. Smooth variations in time and position of *in vivo* force distributions over epithelial regions have been discretised to *in silico* force distributions that vary with time but not with position over epithelial regions. It is worth noticing that the components constituting the *in vivo* mechanism of invagination (mesodermal apical constriction, ectodermal basal constriction, ectodermal and mesodermal radial shortening) have been preserved. (**d**) Time-trends of average radial mesodermal and ectodermal thicknesses of the epithelium. Measurements *in vivo* have been repeated on a set of three different animals of the same genotype, and average trend and error bars (standard error of the mean) are obtained through statistical analysis of this data set (see Methods). It is worth noticing that mesodermal cells character begins differentiating from the ectodermal one approximately at t = −10.43 min (red vertical line), before which all cells share a communal epithelial fate. *In silico*, mesodermal radial thickness M (see schematic in panel e) has been measured at different angles of an angular section of the epithelium, which spans approximately 50 degrees astride ventral point V (25 degrees in each direction from V). In silico measurements for ectodermal radial thickness E, instead, stretched approximately up to 120 degrees astride the dorsal point D (60 degrees in each direction from D). (**e**) Time-trends of furrow's height h at the ventral side (see schematic in panel d).

Our quantification and digitisation of the wild type invagination starts during the fast phase of cellularisation and shows that it finishes in the mesoderm before the ectoderm, with the mesodermal cells beginning to radially lengthen before cellularisation is complete in the ectoderm, see Fig. 3 and Video V1. The cell lengths are quantified in Fig. 3d, which shows that prior to t = −10.43 min mesodermal cells increase their radial length at an average rate of 0.8 µm/min which corresponds to the same value measured by Lecuit et al. [17] for the fast phase of cellularisation. Fig. 3d also shows that the rate of growth of the ectodermal cells during cellularisation is slower than that of mesodermal cells (0.66 µm/min). This is evidence that the origin of differing cell fates is established early, perhaps during the slow phase of cellularisation.

At t = −10.43 min, when mesodermal cells are on average 24 microns long, wild type mesodermal cells increase their radial length at approximately 1.20 µm/min, which is ∼3× that seen for ectodermal cells over this same period. This may be due to the fact that mesodermal cells have begun to actively lengthen, whereas ectodermal cells have yet to complete cellularisation. This hypothesis is supported by the evidence shown in Fig. S1 that epithelial cells consume yolk as they increase in area up until t = 0 min.

At t = 0, the ectodermal cells appear fully cellularised and begin to shorten radially, at an approximate rate of 0.1 µm/min. Just prior to this, at t = −1.8 min, the mesodermal cells reach their peak height, before immediately shortening at a rate of 1.8 µm/min, which is 18–times greater than the rate of ectodermal cell shortening (Fig. 3d).

From observation alone it is not possible to ascertain whether the mesodermal and ectodermal shortening observed is due to an active force generated within the cells or is passive. This because an observed shortening could be due to its neighbour cells lengthening, which would be a passive response, or to the cell itself shortening using its internal cellular mechanisms, which would be an active response. To resolve this issue we used our biomechanical model (Fig. 2) to investigate quantitatively what active cellular forces are required to produce the movements observed. In the model, mesodermal apical constriction, mesodermal shortening, ectodermal basal constriction and ectodermal shortening are all simulated as independent active forces. They can be localised both temporally and spatially within the embryo and switched on and off in any combination.

In previous work we have measured the temporal–spatial distribution of forces during ventral furrow invagination in Drosophila [1]. Here we have discretised these forces so that they could be implemented in a step–wise fashion (as shown in Fig. 3c), rather than in a spatial and temporal continuum. This change from continuous to a discrete set of forces is important for our systematic perturbation analysis and hypothesis testing, which is the focus of this paper.

The mesodermal apical constriction forces were induced to act first. Then at t = −1.23 min mesodermal shortening forces were switched on along with forces causing basal constriction and shortening of the ectoderm, while mesodermal apical constriction were reduced in intensity. The discrete timings and relative forces implemented in this model of ventral furrow formation are shown in Fig. 3c, and shown in Video V2. As can be seen from the experiment (Fig. 3b) this simulation replicates the main features of wild type invagination. Moreover, as shown in Fig. 3, it is also in good quantitative agreement with experiment as measured by two key parameters: the average thickness 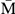 of the mesoderm (Fig. 3d) and height h of the furrow (Fig. 3e); see also Fig. 1 for a schematic view of these parameters. Although the effect of implementing the active shape changes in a discrete manner means that changes occur relatively abruptly in the model, the mesoderm thickness in the model follows a very similar path to that of the experiment (Pearson's 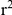 = 0.75, see Table 1) deviating on average a few standard errors from the *in vivo* data (RMSSD = 3.93). Relative differences between the *in silico* and the *in vivo* values in the final invagination configuration amount to less than 5% of 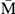 and h, and less than 15% for the average thickness of the ectoderm 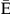 (Fig. 3d–e). As shown in Fig. 3d, within the ectoderm, the model only mirrors the experimental values after it has reached its maximum thickness at t = 0 (Pearson's 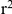 = 0.86) deviating on average only one and a half standard errors from the *in vivo* data (RMSSD = 1.45). Before this time point the *in vivo* and *in silico* data differ possibly because the model does not include growth of the ectoderm associated with on-going cellularisation (for t<0 the ectodermal region in our model already undergoes apical-basal shortening whereas *in vivo*, the apical-basal length of ectodermal tissue is still increasing, see Fig. 3d). This is reflected by a very low Pearson's 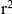 = 0.19, when computed over the invagination interval, although *in silico* predictions still follow quite closely the experimental data (RMSSD = 1.26). Fig. 3e shows that height of the furrow in the simulation matches the height obtained in the experiment very well (Pearson's 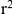 = 0.87), although again the discrete nature of the implementation of the active shape changes causes the height profile to be less smooth than the experiment (RMSSD = 4.26).

**Table 1:** Goodness-of-fit analysis for *in silico* versus *in vivo* curves.

		GOODNESS OF FIT	
		Pearson's 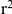	RMSSMD
*wt*	meso	0.75	3.93
	ecto (all t)	0.19	1.26
	ecto (t>0)	0.86	1.45
	height	0.87	4.26
*bnt*	meso	0.95	2.85
	height	0.97	11.90
*arm*	meso	0.85	4.23
*cta/t48*	meso	0.60	0.99

**Figure 4:**
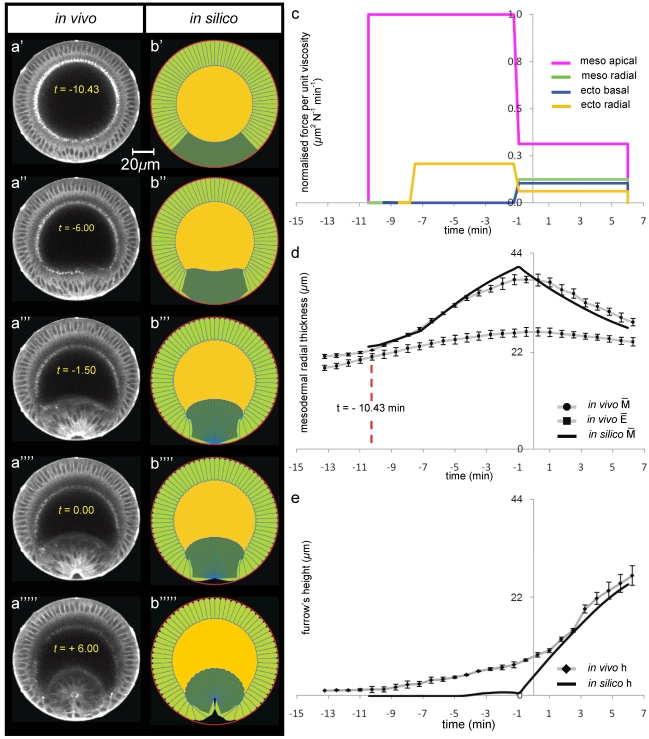
Modelling ventral furrow invagination: *bnt*-mutant genotype. Selected multi-photon images of *in vivo* transverse cross-sections of a *bicoid*,*nano*,*torso*-like mutant embryo during ventral furrow formation. *bnt*-mutant has germ-band extension and posterior mid-gut invagination suppressed. The embryo is labelled with Sqh-GFP and oriented with its dorsal surface upward (Fig. 1a). Time interval between successive frames is 45 sec and instant zero was set by using apical-basal cell height profiles on the dorsal side (Fig. S2b). (**a**) Selected multi-photon images (columns) of *in vivo* transverse cross-sections of a *bnt*-mutant embryo during ventral furrow formation. The embryo is labelled with Sqh-GFP and is oriented with its dorsal surface upward (Fig. 1a). The time interval between successive frames is 45 sec. Time zero was set by using apical-basal cell height profiles on the dorsal side (see panel d). (**b**) Selected frames (columns) of *in silico* transverse cross-section of a *bnt*-mutant genotype. Embryo's 2D geometry is oriented with its dorsal side upward. The undeformed initial configuration (panel b′) corresponds to *in vivo* deformations at t = −10.43 min (see panel d). (**c**) *In silico* force distributions utilised to simulate invagination in b). (**d**) Time-trends of average radial mesodermal (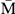) and ectodermal (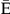) thicknesses of the epithelium (see schematic in Fig. 3e). (**e**) Time-trends of furrow's height h at the ventral side (see schematic in Fig. 3e).

**Figure 5:**
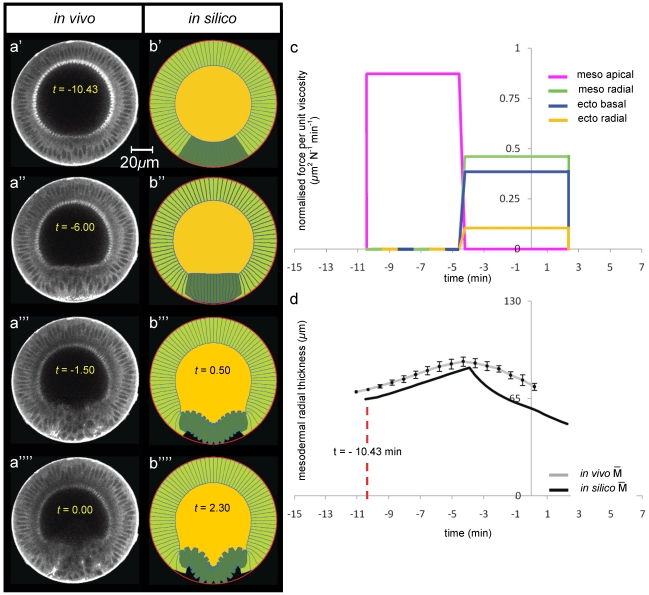
Modelling ventral furrow invagination: *armadillo*-mutant genotype. (**a**) Selected multi-photon images of *in vivo* transverse cross-sections of an *armadillo*-mutant embryo during ventral furrow formation. *armadillo*-mutant lacks strong apical junctions, and ventral indentation collapses at t = −4.22 min. The embryo is labelled with Sqh-GFP and oriented with its dorsal surface upward (Fig. 1a). The time interval between successive frames is 45 sec and instant zero was set by using apical-basal cell height profiles on the dorsal side (Fig. S2c). (**b**) Selected frames (columns) of *in silico* transverse cross-section of an *arm*-mutant genotype. Embryo's 2D geometry is oriented with its dorsal side upward. The undeformed initial configuration (panel b′) corresponds to *in vivo* deformations at t = −10.43 min (see panel d). (**c**) *In silico* force distributions utilised to simulate invagination in b). (**d**) Time-trends of average radial mesodermal (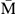) thickness of the epithelium (see schematic in Fig. 3e).

**Figure 6:**
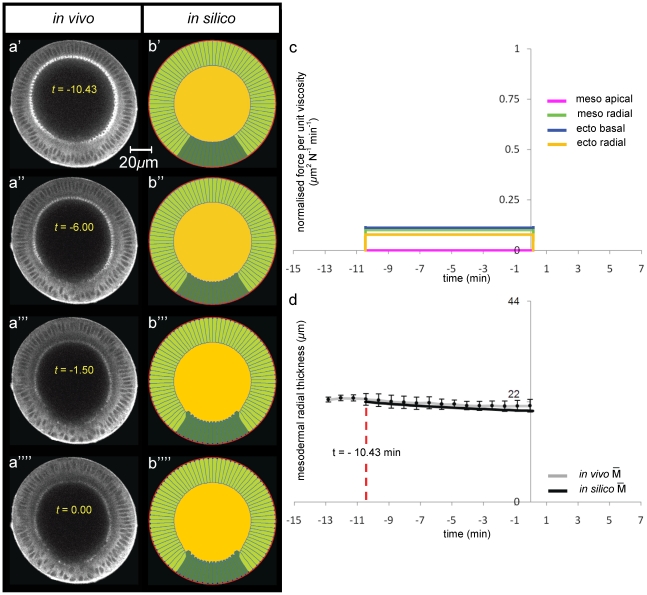
Modelling ventral furrow invagination: *cta/t48*-mutant genotype. (**a**) Selected multi-photon images of *in vivo* transverse cross-sections of a *cta/t48*-mutant embryo during ventral furrow formation. In *cta/t48*-mutants apical constriction is completely abolished. Embryo is labelled with Sqh-GFP and oriented with its dorsal surface upward (Fig. 1a). Time interval between successive frames is 45 sec and instant zero was set by using apical-basal cell height profiles on the dorsal side (Fig. S2d). (**b**) Selected frames (columns) of *in silico* transverse cross-section of a *cta/t48*-mutants genotype. Embryo's 2D geometry is oriented with its dorsal side upward. The undeformed initial configuration (panel b′) corresponds to *in vivo* deformations at t = −10.43 min (see panel d). (**c**) *In silico* force distributions utilised to simulate invagination in b). (**d**) Time-trends of average radial mesodermal (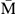) thickness of the epithelium (see schematic in Fig. 3e).

*In silico* predictions for *wt* as well as for *bnt*, *arm* and *cta/t48* mutant embryos have been compared to respective *in vivo* trends (Figs. 3, 4, 5, and 6) by using Pearson's 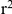 and RMSSMD (root mean squared scaled deviation) measures of goodness-of-fit [26] for the mesoderm (meso), the ectoderm (ecto) and the furrow's height (height). The closer 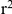 is to 1, and the closer RMSSMD is to zero, the closer the *in silico* predictions match the *in vivo* dataset. For the wild type, ectodermal goodness-of-fit has been analysed for all time instants in the experimental invagination interval (Fig. 3d–e) as well as for t>0 minutes only.

### Agreement between mutant phenotypes of simulation and experiment validates the model

A more stringent test of this biomechanical model is to use it to predict mutant phenotypes lacking in one or more of the force components actively involved in invagination, and to compare these quantitatively with experimental data. For this analysis, we used three mutants: *bnt*, to test the role of forces elsewhere in the embryo; *arm*, to explore the role of cell–cell adhesion and the transmission of tension; and *cta/t48* to test the role of apical constriction itself. We consider each in turn.

The *bnt* mutant lacks AP patterning while dorsal–ventral patterning remains intact. During invagination mesodermal average radial thickness increases at an approximate rate of 0.7 µm/min until t = −10.43 min, after which it starts increasing at the higher rate of 1.3 µm/min (Fig. S2). It is worth noting that at approximately t = −7.5 min, the *bnt* mesodermal cells start lengthening at an even faster speed of approximately 2.3 µm/min, a rate almost double that of *wt* mesodermal cells (Fig. 4d and Video V3). After this the *bnt* mesodermal cells shorten radially with a constant rate of approximately 1.3 µm/min until invagination is complete, which is almost 30% less than the rate seen in *wt* mesodermal cells (Fig. 4d). In the ectoderm, cellularisation proceeds at approximate rates of 0.9 µm/min and 0.5 µm/min respectively before and after t = −10.43 min, until cells reach a maximum height at t = 0 min. Ectodermal cells then shorten at an average speed of 0.2 µm/min, which is double that of the *wt* embryo.

The *bnt* mutant was modelled by increasing the width of the active mesoderm to a region comprising 84 degrees astride the ventral point (the mesoderm in all other simulations covers 68 degrees across the ventral point – see Fig. 1). This value is taken from experimental data which shows that Myosin II that is initially localized on the basal side of the epithelium (red–dotted line in Fig. 1a) vanishes, while myosin newly appears on the apical side (on the opposite end of cells) in a region that in the *bnt* spans a wider angle (84 degrees) than in the *wt* embryo (64 degrees) as shown in Fig. 1. When t = −7.5, mesodermal cells in *bnt* mutants lengthen at a faster rate and to a greater extent than mesodermal cells in *wt* animals. This phenomenology can only be reproduced *in silico* either by introducing an active lengthening of mesodermal cells at t = −7.5 or by advancing ectodermal shortening at t = −7.5. Since there is no plausible biological mechanism in the literature in support of the first option, the onset of ectodermal radial shortening in the model was advanced to t = −7.5 s with respect to the *wt* embryo model, doubled in intensity in the first invagination interval and reduced in the second interval of invagination (Fig. 4c). This also correlates to experimental evidence showing the distribution of myosin–II at the basal end of ectodermal cells (Fig. 1a and Video V4).

Comparison of Fig. 4a and 4b shows the evolution of the *in silico bnt* phenotype which compares very well the experimentally obtained phenotype. Fig. 4d shows that both the increased mesodermal thickness profile, and the decreased furrow height profile of the *bnt* mutant are matched by the simulation excellently in terms of trend, with Pearson's 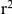 = 0.95 for mesodermal trend and Pearson's 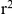 = 0.97 for furrow's height curve. The discrete nature of the force profiles still affects the *in silico* path with RMSSD = 2.85 and 11.9, for the mesodermal and furrow path respectively. The relative difference between the *in silico* and the *in vivo* values in the final invagination configuration amounts to less than 4% for 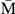 and less than 4% for h (Fig. 4d–e). Importantly this data shows that the experimental trends of increased mesodermal width and lower furrow height are mechanically necessary and coupled.

Embryos mutant in *arm*, which lack β–catenin, an essential component of adherence junctions between epithelial cells, initially behave like *wt* embryos, with the mesodermal cell apically constricting as normal, and the furrow starting to form, see Fig. 5a. However at t = −4.22 min the cell–cell apical junctions fail and the arching furrow collapses back into contact with the vitelline membrane, see Fig. 5b. As a consequence for t>1 min the cells start rounding up and make digitisation process difficult. At times later than 6 min, the mesoderm buckles into several folds (Video V5).

The *arm* mutant was modelled by turning off mesodermal apical constriction at t = −4.22 min, once the furrow rises up from the vitelline membrane to simulate the inability to transmit tension between the cells. Fig. 5c shows the force profile, and Fig. 5b illustrates the development of the phenotype during the simulation which shows good qualitative agreement with experiments. Fig. 5d shows the quantitative comparison of the mesoderm thickness between the experiment and the simulation. Agreement with the experimental trend over time is very good (Pearson's 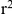 = 0.85), with the relative difference between the *in silico* and *in vivo* values in the final instant of invagination amounting to 34% for 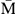. The *in silico* predictions still deviate on average a few standard errors from the *in vivo* data– due to discrete nature of force profiles utilised (RMSSD = 4.23). The data clearly shows the extent to which model struggles to reproduce quantitatively the *in vivo* deformations despite giving good qualitative agreement (Video V6).

Embryos doubly mutant for *cta* and *t48* do not form any visible ventral furrow (Video V7). We see a very similar behaviour in our *cta/t48* double mutant embryos; the mesoderm does not apically constrict or thicken, and there is no furrow formation, see Fig. 6a. The *cta/t48* double mutant is modelled by turning apical constriction off in the model. Fig. 6b shows the development of the phenotype during the simulation, which shows good qualitative agreement with experiment when comparing the radial thinning undergone by the mesodermal regions. The quantitative comparison shows a very acceptable Pearson's 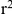 = 0.60 and RMSSD = 0.99, with the relative difference between the *in silico* and the *in vivo* data in the final invagination configuration amounting to less than 8% for the 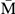 (Fig. 6d and Video V8).

### Ventral furrow formation is robust to large biomechanical perturbations

**Figure 7:**
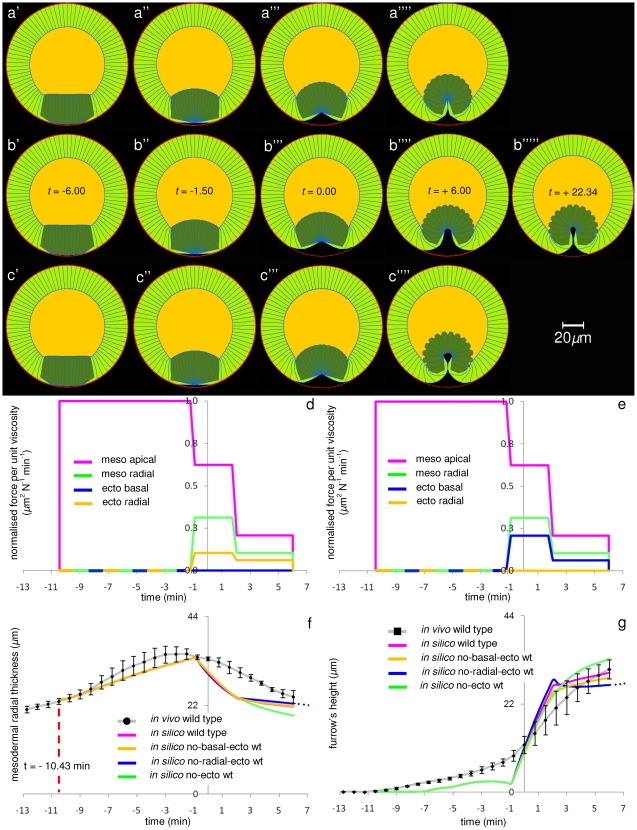
Ventral furrow invagination robustness to *in silico* ectodermal perturbations. The wild type mechanism of invagination (Fig. 3c) has been perturbed by switching on/off component forces acting in the ectoderm (blue and yellow lines). The figure shows that perturbing the wild type mechanism of invagination does not hinder ventral furrow formation, which is ultimately regained in a shorter or longer period depending on the typology of perturbation. It is worth noticing that the perturbation introduced in the invagination mechanism (see panels f–g) does not affect phenotypes corresponding to t<−1.5 min, which remain the same as in the wild type case (Fig. 3a′–a″). (**a**) Phenotypes relative to the wild type mechanism where no basal ectodermal constriction is activated. (**b**) Phenotypes relative to the mechanism where no radial ectodermal shortening has been activated. (**c**) Phenotypes relative to the mechanism where both ectodermal movements have been turned off, resulting in an inactive ectoderm. (**d**) Force trends utilised to simulate the wild type invagination mechanism with no ectodermal basal constriction – the component that corresponds in the graph to this movement has been suppressed (blue line). (**e**) Force trends utilised to simulate the wild type invagination mechanism with no ectodermal radial shortening – the component that corresponds in the graph to this movement has been suppressed (yellow line). Similarly, force trends to simulate wild type invagination with no active ectodermal forces (both basal and radial ones) have been obtained by suppressing the lines that correspond in the graph to these two movements. (**f**) Time-trends of average radial mesodermal (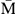) thickness of the epithelium (see schematic in Fig. 3e). (**g**) Time-trends of furrow's height h at the ventral side (see schematic in Fig. 3e). The blue dotted lines in panels (f) and (g) indicate that the simulation detailed in (b) (where no radial ectoderm has been activated) fully invaginates on a longer period of time with respect to that shown in figure panels.

**Figure 8:**
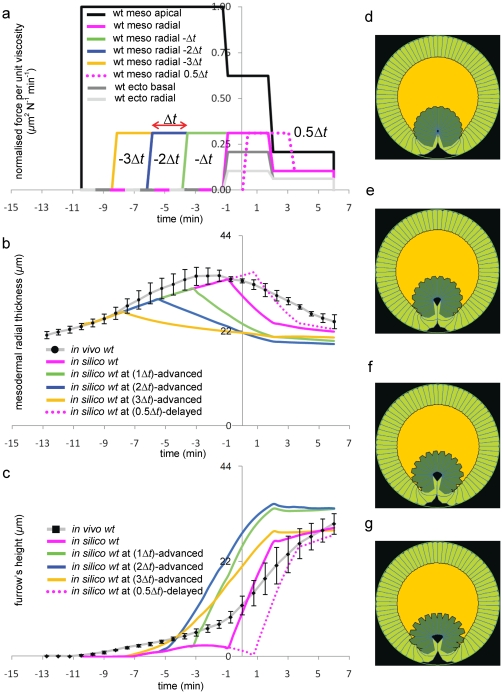
Meso-radial time study. The quantitative effects of anticipating the onset of mesodermal radial shortening with respect to the wild type case reported in Fig. 3, while keeping the remaining force trends unchanged (Fig. 3c). (**a**) Force trend curves labelled by 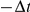, 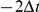, 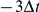 and 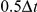 illustrate the case where meso-radial movement was respectively advanced at t = −3.48 min, t = −5.8 min, t = −8.12 min and delayed at t = 0.58 min with respect to the *wt* case (where meso-radial movement onsets at t = −1.2 min, as shown in Fig. 3c). (**b–c**) Changes in the onset time of this movement with respect to the others have significant impact on furrow's height h, which increases with the anticipation of the movement. The average mesodermal thickness 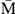 decreases with the anticipation of the movement in that mesodermal cells start shortening earlier. (**d–g**) Final phenotypes (t = 6 min) corresponding to wild type with mesodermal radial movements respectively delayed by 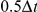 (dotted lines in panels a–c) and advanced by 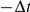, 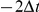 and 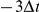.

To test the robustness of the invagination mechanism to the loss of ectodermal forces we carried out a set of simulations in which we perturbed them systematically. In the first set of simulations we removed ectodermal basal forces, then ectodermal radial shortening forces, and finally all ectodermal forces. Fig. 7a, b, c shows the phenotypes obtained, and Fig. 7d–e shows the timing–force diagrams for the new mechanisms tested. The results show that while ectodermal forces are not essential for furrow formation they do affect the shape of the furrow and the speed at which is it developed. Fig. 7f–g shows that ectodermal forces have very little effect on mesodermal thickness and furrow height. But Fig. 7b–c show that the loss of ectodermal radial shortening causes a hole to be formed in the furrow (a feature sometimes observed *in vivo*); a similar result is obtained by increasing or anticipating shortening of mesodermal cells (Fig. 8e–g). We showed that the mechanism is robust to ectodermal radial timing perturbations (Fig. S3), ectodermal basal timing perturbations (Fig. S4), ectodermal radial intensity perturbations (Fig. S5) and ectodermal basal intensity perturbations (Fig. S6).

Fig. 8 shows the effect of perturbing the onset of mesodermal radial shortening with respect to the wild type case. The effect is quite dramatic, with both the thickness of the mesoderm and the height of the furrow affected, however invagination still completes, with the final phenotype relatively unaffected (Fig. 8d–f). The effect of changing the intensity of the mesodermal radial shortening forces dramatically affects both mesodermal thickness and the height of the furrow, however invagination still completes (Fig. S7). In contrast reducing or increasing the intensity of mesodermal apical constriction has very little effect on the height of the furrow or the thickness of the mesoderm as well as on the shape of the furrow (Fig. S8).

## Discussion

Both the experiments and the biomechanical model confirm that the mesodermal forces are crucial for ventral furrow invagination, as the *arm* and *cta/t48* mutants show, loss of apical constriction leads to a loss of the furrow. The biomechanical model confirms that even if the active mesodermal radial shortening and the ectodermal forces still operate, they are not enough to restore the furrow in the absence of mesodermal apical constriction, as shown for the *cta/t48* mutant, see Fig. 6.

Mesodermal radial shortening forces are also shown to be critical to furrow formation. Previous *in silico* predictions by Pouille et al. [18] suggested that both apical-basal cell elongation and shortening during invagination were passive mechanical consequences of the active process of increasing the apical membrane tension and flattening in invaginating mesodermal cells. Our previous Video Force Microscopy analysis [1] – in accordance with previous experimental hypotheses by Leptin [19] and Costa et al. [4] – provided direct evidence that significant tension along the lateral edges of presumptive mesodermal cells assists internalization of apically constricted cells by shortening them. Biomechanical analysis presented in this paper confirms that mesodermal radial shortening forces are the primary cause of the rise of the furrow in the model and so strongly affect furrow height whereas mesodermal apical constriction affects this very little. The relative timing of these two active forces also affects the furrow in terms of its height and thickness of the mesoderm (Fig. 8), which raises the question, what signal activates mesodermal radial shortening?

It is significant that Zhou et al. [20] have measured tissue stiffness of amphibian and echinoderm gastrula–stage embryos and found high embryo–to–embryo variability. They comment that this implies that both passive physical and mechanoregulatory processes will determine how sensitive gastrulation is to tissue mechanics. Our biomechanical model does not explicitly simulate such active mechanoregulatory processes, but it confirms that in the case of invagination, the system appears robust with respect to variations of the uncoupled driving forces. That result does not preclude the existence of mechanoregulatory or other regulatory mechanisms involved in coordinating the coupling between mesodermal apical constriction and mesodermal radial shortening. In our model the two follow each other in a preprogrammed sequence but this need not be the case: regulation through mechanical feedback has been experimentally proved to contribute to the process of mesoderm invagination [21] and proposed to account for other aspects of Drosophila gastrulation [22], [23].

The quantitative comparison between the experiments and the biomechanical model indicate that ectodermal forces play a minor but significant role in furrow formation. Without additional mechanisms, the furrow does not close properly and forms a hole in its centre, see Fig. 7. Our analysis shows that closing of this hole in the wild type could be explained by ectodermal radial shortening forces, which squeeze the furrow shut, since absence of this active force also results in a hole. Our previous biomechanical studies of this phenomenon in *Drosophila* did indicate that ectodermal pushing forces had the potential to be a potent force in generating a furrow that is robust to perturbations [10], [24], and so the existence of this force is not entirely surprising. Other workers have also suggested presence of this force [1], [2], [4], here we have examined its role and importance and found that the mechanism appears to be very robust to both temporal and spatial ectodermal perturbations. Sherrard et al. [14] have found no role for ectodermal forces in *Ascidian* invagination whereas Butler et al. [21] have proposed that cell shape changes contributing to germ-band extension are a passive response to mechanical forces caused by mesodermal invagination. Although it remains to be seen how widespread ectodermal forces are used for robust invagination in other biological organisms, our discovery that to simulate the *bnt* mutant requires a greater role for ectodermal forces (Fig. 4) may provide evidence that other morphogenetic mechanisms have a biomechanical impact on the ventral furrow formation. The cephalic furrow is absent in *bnt* mutants as is the posterior midgut (PMG), which internalizes at the posterior dorsal side at the time the furrow is formed in wild type. Moreover, there is no germ band extension in *bnt* mutants, which could also be significant since it starts at the same time as the furrow is formed in wild type.

This experiment therefore tests whether the model is also able to recapitulate the movements in the absence of external morphogenetic effects. However it should be noted that as a result of the loss of the cephalic furrow, the ventral furrow in *bnt* embryo is longer than in the wild type embryo and so the position of the section studied along the length of the embryo is slightly different relative to the wild type, which accounts for wider active mesodermal region. A contributing factor in the absence of the cephalic furrow, PMG and germ band extension, could be an increase in yolk pressure on the ventral epithelium which may be significantly different causing a greater lengthening of the mesodermal cells and perhaps accounting for the greater need of the ectodermal forces to balance these. A 2D hydrodynamic model proposed by Pouille and Farge [12] predicted the need for a radial centripetal force in order to achieve the complete internalisation of the furrow. They postulated this force to be due to the curvature that in 3D characterises in vivo embryos along the anterior-posterior axis. While we in our 3D model [9] and others [15] have not found evidence for the influence of passive forces generated elsewhere in the embryo on the internalisation of the furrow, clearly further work needs to be done to fully investigate possible secondary effects due to the 3D nature of the embryo.

It is becoming increasingly clear that actin–myosin based cell shape changes, such as apical constriction occur in a pulsed manner [25]. In our study the time scale of our measurements is too large to resolve pulses and our stepwise to model which increases and decreases the mesodermal apical constriction force is not intended to simulate such behaviour, for the same reasons. Although we intend in future work to resolve the forces at a finer scale in the model and experiment, it is beyond the scope of the current work, which was to perform a systems biomechanical analysis to analyse which combination of active forces determines the invagination geometry, and to reveal the effect of relative timing of different active forces.

In this paper we have successfully shown the self–consistency of the model in that it does not require any fitting parameters to move from simulating the wild type to simulating the mutants, all that changes are the relative timings and magnitudes of the forces which we explicitly specify. Such checks for self–consistency give us confidence that the model is able to help us understand the biomechanical system of ventral furrow formation. Furthermore we have shown that to understand the role of different forces in ventral furrow formation a quantitative comparison between experiment and model is required, which we have also succeeded in accomplishing. This gives us great confidence in assessing the roles of different active shape changes in the mechanism of ventral furrow formation. Some of the mismatch in Fig. 3, 4, 5, and 6 between our *in silico* model and experiments is undoubtedly due to the time and spatial discretisation of the forces. These ‘errors’ between *in silico* and *in vivo* trends/phenotypes may be reduced by fine tuning input forces utilized to deform the 2D model geometry. Nevertheless, this would not change the conclusions of this study on the robustness mechanism and the role of each force.

We conclude that the mechanism of ventral furrow formation relies on mesodermal apical constriction, followed by (and perhaps triggering) mesodermal radial shortening, ectodermal radial shortening and ectodermal basal constriction. This role for ectodermal forces is in agreement with those proposed other studies of ventral furrow formation [2], [4], [10], [23]. These findings establish the fundamental biomechanical system of forces responsible for robust ventral furrow formation in *Drosophila*.

## Materials and Methods

### Embryos and genotypes

At mid–cellularisation stage, embryos were de–choryonated 2 minutes at room temperature in 50% bleach, followed by several washes with PBS. They were kept in PBS for several hours without apparent developmental delays or problems.

To genetically abolish anterior–posterior polarity, germ–band extension and posterior midgut invagination, we used the stock w; Sqh–GFP^42^; *bicoid* ^E1^ *nanos* ^L7^ *torso–like^146^*/TM3 Sb and analyzed progeny from mothers homozygous for *bicoid* ^E1^ *nanos* ^L7^ *torso–like^146^* (in short: Sqh–GFP; *bnt* embryos). To specifically block apical constriction, we used *w*, Sqh–GFP^42^; *cta* ^R10^/*CyO*; *Df(3R)^CC1.2^*/TM3 Sb stocks. The Df(3R)CC1.2 deletion uncovers the *t48* locus. 25% of progeny from homozygous *cta^R10^* parents showed a complete absence of apical constriction and were thus assumed to be Sqh–GFP; *cta, t48* embryos. We generated armadillo germ–line clones using the FLP–DFS system^2^. By crossing *arm* ^043A01^ FRT101/FM7; Sqh–GFP^42^ females to *w, ovo^D^*, FRT^101^/Y; flp–138 males, we obtained arm^043A01^ FRT^101^/*w, ovo^D^*, FRT^101^; flp–138/+ females. These females were heat shocked as larvae for 2 hr at 37°C to induce mitotic recombination in the germ–line and crossed to FM7/+; flp–138/+ males.

### Multi–Photon Microscopy

Transgenic Sqh–GFP embryos were mounted vertically in 1% agarose in PBS on Mattek culture dishes, with the posterior end facing the objective. They were imaged on a custom–built two–photon microscope, using a Nikon 40× NA 0.8 objective and simultaneous epi– and trans–detection as described in ^3^. We used laser lines between 890 and 910 nm. A 488 nm laser with an output power of 15W generated 150 mW laser pulses between 890 and 910 nm at the level of the objective. Embryos imaged under these conditions were able to develop normally afterwards. Imaging depth was 110 µm from the posterior end of the embryo, and images were acquired every 45 seconds with MATLAB. Note that the furrow is wider in *bnt* embryos.

### Synchronization of Embryos and *in vivo* data analysis

A set of three different animals of the same genotype has been quantified for each genotype in order to obtain all microscopy data shown in this work. In this work we utilised a timer to synchronise time evolution of all the embryos analysed. According to this timer, the origin of time (instant zero, t = 0) is set for each embryo at the point of average maximal radial growth of ectodermal cells, see Fig. 3d. Importantly, this proved a reproducible timer that was independent of events occurring within the ventral furrow [1]. Coresponding *in vivo* trends have been obtained through statistical analysis of the resulting data set, where error bars are given by standard error of the mean. Since *in silico* predictions can fit *in vivo* trends quite well and yet completely miss the exact locations of the data – and vice versa – we also computed goodness-of-fit of *in silico* vs. *in vivo* datasets by using both Pearson's 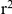 test and RMSSD measures (root mean squared scaled deviation) [26], see Table 1. The former assesses how well the *in silico* curve captures the relative *in vivo* trend whereas the latter estimates deviation of our *in silico* predictions from exact locations of *in vivo* data points: the quantity 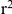 varies in the interval [0,1] and the closer 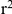 is to 1 the better the agreement [26]. The quantity RMSSD is obtained by scaling absolute deviation of each *in vivo* data point by the standard error of the data and quantifies how close the model is to the *in vivo* data points (the lower RMSSD the better the agreement) [26].

### Digitisation of embryo movies

Custom software was used to generate a reference mesh that was overlaid and adjusted manually to fit the first image of each movie of the embryo. Automated and manual methods were used to position the registration points of the mesh to track the *in vivo* movements from image to image in each set. This included ccustom software to enable multiple frames to be viewed in both forwards and backwards directions so that the location of the registration nodes could be placed with precision. A combination of automated and manual methods was used to track the fiduciary points from image to image when the edge of the cell was difficult to discern. Image contrast was locally optimized in each frame with image processing techniques (using Adobe Photoshop) in order to bring out contour of the less visible parts of the forming furrow. Placement of fiduciary points in difficult areas of the *in vivo* images was also further assisted by automatic constant–volume algorithms emulating conservation of cell cytoplasmic volume. Combination of these two techniques and high level of image zooming ensured tracking error was to same level as for the clearest parts of the images.

Reference configuration for deformations of all embryos is set at 10.43 minutes before instant zero (i.e. t = −10.43 min, see above for temporal synchronisation of embryos). An angular coordinate system was also defined at t = −10.43 min on the circular reference configuration of each embryo in order to locate specific areas of the epithelium. The presumptive mesoderm is defined as the tissue that ultimately forms the ventral furrow. By tracking backward in time, this tissue was found to be nominally 18 cells wide in the *wt* embryo. In the wild type and *bnt* mutant embryos, this sub–region was respectively found to span approximately ±30° (green lines) and ±40° (blue lines) astride the dorsal–ventral midline. Fig. 1b shows the mesh of registration points utilised for the digitisation procedure of an intermediate–stage frame from the *in vivo* imaged sequence of a *wt* embryo: the polygonal mesh (blue lines) has been overlaid on the tissue to track displacements at registration nodes (magenta dots). Polygonal partitions can correspond to single cells, multiple cells or sub–cellular regions, depending on the measurements refinement required in that particular portion of tissue. Yellow and green arrows on Fig. 1b indicate the radial thickness of the epithelium, which was measured on the mesh as the distance between basal and apical node along the same side of a given cell. The height of the furrow (h) (red arrow on Fig. 1b) has been measured as the radial displacement of the most ventral nodal point from its position in the reference frame. Movies of the wild type and mutant embryo can be seen in the Supplementary Material.

### Biomechanical Model

The simulations are based on a finite element method which involves modelling the key biomechanical features of cells and the embryo structure, see Fig. 2. In order to build a finite element model of the single epithelial cell, the effects of the complex force fields originating from molecular processes were assumed to be resolved into equivalent forces along cell edges (Fig. 2 a–c). The incompressibility of the inner cytoplasm was simulated by enforcing a constraint that the surface region enclosed by the cell membrane remained constant over time (Fig. 2c–d). Cell–sized regions of the embryo cross–section were partitioned into 5 quadrilateral elements with nodes at the corners of each element (Fig. 2d). A radial subdivision is used to allow individual cells to bend, as they do *in vivo*. In keeping with current models of morphogenetic movements in embryos [1], [13], [27], [28], the cytoplasm was assumed to be viscous, and its effective viscosity was assumed to arise from the mechanical properties of passive cellular components such as the cytosol and organelles. The forces 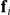 needed to drive incremental nodal displacements 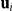 over time increment 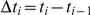 were discretised in time with an explicit algorithm yielding the following set of equations:
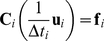
(1)where 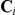 is a so called damping matrix. It parallels the more common stiffness matrix, but is applicable to viscous materials as opposed to elastic ones. To understand the physical meaning of Eqn. (1), consider a small, not necessarily rectangular, block of viscous material and assume that small incremental displacements 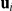 of its corners are applied over a short time increment 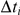. The forces 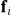 that would need to be applied to the corners are well defined and they depend on the geometry of the block and the viscosity of its material: they are given by Eqn. (1) [29]. Eqn. (1) can also be used together with appropriate boundary conditions to determine the incremental displacements that would occur if specified nodal forces were applied, or it can be used to relate various combinations of forces and displacements to each other, provided that the resulting system of equations is well posed. In a finite element model, equations like (1) are written for each element and they are mathematically assembled together to give global systems of equations that describe how all of the nodal forces and displacements are related to one another. In the present context, this set of equations relates the viscous forces in the cells in the cross–section shown in Fig. 2e to the incremental motions that they undergo.

In our previous inverse analysis [1], the displacements 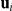 are retrieved from *in vivo* images, and the set of driving forces 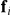 that satisfy Eqn. (1) were computed. Instead, in the forward analysis used here, a set of forces are applied, and the associated displacements 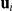 are computed by solving Eqn. (1). We emphasize that for the purpose of the robustness analysis, the forces employed in our forward analysis have a simpler profile than those retrieved in the inverse analysis.

The active components of the cell include microfilaments and microtubules, and they are assumed to generate forces that can be resolved along the edges of the finite elements [28], [30]. Forces along edges that share a common boundary are added together, and applied vectorially to both of the common nodes, see Fig. 2d. In the case of cells containing multiple finite elements, forces along the internal edges are assumed to be zero. When the driving forces acting during a particular time step 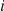 are added together node by node, they produce a system of driving forces 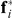. For the embryo cross–section to be in a state of dynamic equilibrium, these driving forces 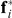 must just equal the forces 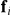 generated by deformation of the cytoplasm as a result of incremental displacements 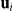. Thus, at each incremental step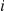, the forces 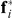 are calculated based on the current geometry, they are substituted into the right hand side of Eqn. (1), and the equation is solved for the incremental nodal displacements 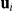. These displacements describe node–by–node how the embryo cross–section would change from one moment to the next if acted on by the system of forces 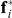. In this way, one can use the model to determine the changes in embryo shape that would result from any user–specified set of driving forces 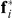. The embryo simulations were analysed in the same way as the experiment and movies of the simulations were generated for visualization.

The vitelline membrane has been modelled by introducing a rigid boundary condition that prevents apical nodes from moving outside a bounding circle (Fig. 1b and Fig. 2e). Consequently, the sum of the cross–sectional areas of the yolk, epithelium cells and ventral gap (the region where cells pull away from the vitelline membrane) is constrained to be constant. The yolk was modelled as a compressible viscous fluid exerting a pressure on the inner (basal) side of the epithelium to a maximum of 5% of its initial value. The experimental value of the yolk pressure is not known, so this was a system variable.

### Invagination forces, viscosity and dynamics

For each cell the viscosity is used to define the damping matrix 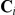 in Eqn. (1), which sets the magnitude of the forces operating. The problem from a modelling stand point is that the experimental values of viscosities available in the literature vary by eight orders of magnitude in the interval from 6.8×10^−17^ N min (µm)^−2^ (i.e. 4.3×10−3 Pa.s, as in Gregor et al. [31]) to 1.7×10^−9^ N min (µm)^−2^ (i.e. 10^5^ Pa.s, as in Bittig et al. [32]). This problem can be circumvented by normalizing the range of forces ‘per unit of viscosity’ that quantitatively reproduced *in silico* the invagination path observed *in vivo*. The units of these normalised forces are µm^2^ N^−1^ min^−1^, and so forces measured in Newton are recovered simply by multiplying by a specific viscosity value. This approach does not change the location, timing or relative magnitude of the forces – the factors central to this systems analysis.

For the purpose of our perturbation analysis, we transformed the forces obtained from our VFM study [1] from a continuous to a discrete set that could be turned on and off. Once the model had been tuned in this way for the wild type simulation, see Fig. 3c, the range of forces were normalised and the same constants were used for all other mutant simulations and the perturbation analysis. In other words none of the parameters were changed when we used the model to simulate the mutant prototypes, the only things that change are the relative forces and timings, which were obtained from experiment and are shown in the figures.

## Supporting Information

Figure S1 ***In vivo*** **epithelium and yolk surface trends.** Epithelium and yolk area trends versus time in different genotypes (one animal per genotype). Embryos were synchronized at t = 0 min using apical-basal cell height profiles on the dorsal side (Fig. 3). The average maximal extension of dorsal cells (t = 0 min) occurs at the maximal area of epithelium and minimal area of the yolk, which is a good indicator for the end of cellularisation of ectodermal cells. (**c**) cell-cell apical junctions in the *arm* mutant disrupt at t = −4.22 min (green vertical line), thus leading to the collapse of the ventrally denting furrow.
(TIF)

Figure S2 ***In vivo*** **mesodermal and ectodermal radial thicknesses.** Mesodermal and ectodermal trends versus time representing radial thicknesses across an angular span of tissue astride the ventral (V) and dorsal (D) points respectively. Measurements refer to a single animal per genotype. Mesodermal trends refer to radial thicknesses across an angular span of approximately 50 degrees astride ventral point V (25 degrees in each direction from V, figure 1a), whereas ectodermal trends refer to radial thicknesses across an angular span of approximately 120 degrees astride the dorsal point D (60 degrees in each direction from D, figure 1a. In vivo *wt*, *bnt*, *arm* and *ct/t48* embryos were synchronized at t = 0 minutes by averaging the values of dorsal ectodermal cell length at each instant and aligning the maxima of such values for each genotype. (**c**) Cell-cell apical junctions in the *arm* mutant disrupt at t = −4.22 min (green vertical line), thus leading to the collapse of the ventrally denting furrow.
(TIF)

Figure S3
**Ecto-radial time study.** The quantitative effects of anticipating the onset of ectodermal radial shortening with respect to the wild type case reported in Fig. 3, while keeping the remaining force trends unchanged (Fig. 3c). (**a**) Force trend curves labelled by 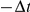, 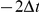 and 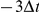 illustrate the case where ecto-radial movement was respectively advanced at t = −3.48 min, t = −5.8 min, t = −8.12 min with respect to the *wt* case (where ecto-radial movement onsets at t = −1.2 min, as shown in Fig. 3c). (**b–c**) changes in the onset instant of this movement with respect to the others has no significant effects on furrow's height h but impacts mesodermal rate of thickening, which increases with the anticipation of the movement. (**d–f**) Final phenotypes (t = 6 min) corresponding to wild type with ectodermal radial movements advanced respectively at 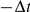, 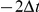 and 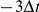.(TIF)

Figure S4
**Ecto-basal time study.** The quantitative effects of anticipating the onset of ectodermal basal constriction with respect to the wild type case reported in Fig. 3, while keeping the remaining force trends unchanged (Fig. 3c). (**a**) Force trend curves labelled by 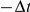, 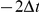 and 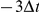 illustrate the case where ecto-basal movement was respectively advanced at t = −3.48 min, t = −5.8 min, t = −8.12 min with respect to the *wt* case (where ecto-basal movement onsets at t = −1.2 min, as shown in Fig. 3c). (**b–c**) changes in the onset time of this movement with respect to the others has significant effects on both mesodermal thickening ratio and furrow's height, which decrease with the anticipation of the movement (with the exception of yellow h trend due to numerical instabilities). (**d–f**) Final phenotypes (t = 6 min) corresponding to wild type with ectodermal basal movements advanced respectively at 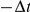, 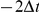 and 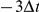.(TIF)

Figure S5 **Ecto-radial intensity study.** The quantitative effects of varying the intensity of ectodermal radial shortening in the time interval [−1.2 min,2 min] (second invagination interval, Fig. 3c). (**a**) Ecto-radial forces were increased/decreased by 25% and 50% of their value in the wild type case (Fig. 3c). (**b–c**) Different simulations refer to an increase/decrease of 25% and 50% in intensity. The perturbation of the intensity of apical constriction in the time interval does not substantially affect either the mesodermal/ectodermal thickness or the height of the furrow in the whole interval of invagination. (**d–e**) Final phenotypes (t = 6 min) corresponding to wild type with ectodermal radial intensity decreased respectively of 50% and 25%. (**f–g**) Final phenotypes (t = 6 min) corresponding to wild type with ectodermal radial intensity increased respectively of 50% and 25%.
(TIF)

Figure S6 **Ecto-basal intensity study.** The quantitative effects of varying the intensity of ectodermal basal constriction in the time interval [−1.2 min,2 min] (second invagination interval, Fig. 3c). (**a**) Ecto-basal forces were increased/decreased by 25% and 50% of their value in the wild type case (Fig. 3c). (**b–c**) Different simulations refer to an increase/decrease of 25% and 50% in intensity. The perturbation of the intensity of basal constriction in the time interval does not substantially affect either the mesodermal/ectodermal thickness or the height of the furrow in the whole interval of invagination. (**d–e**) Final phenotypes (t = 6 min) corresponding to wild type with ectodermal basal intensity decreased respectively of 50% and 25%. (**f–g**) Final phenotypes (t = 6 min) corresponding to wild type with ectodermal basal intensity increased respectively of 50% and 25%.
(TIF)

Figure S7 **Meso-radial intensity study.** The quantitative effects of varying the intensity of mesodermal radial shortening in the time interval [−1.2 min, 2 min] (second invagination interval, Fig. 3c). (**a**) Meso-radial forces were increased/decreased by 25% and 50% of their value in the wild type case (Fig. 3c). (**b–c**) Different simulations refer to an increase/decrease of 25% and 50% in intensity. The perturbation of the intensity of meso-radial forces in the time interval substantially affects both mesodermal thickness and height of the furrow in the whole interval of invagination. (**d–e**) Final phenotypes (t = 6 min) corresponding to wild type with mesodermal radial intensity decreased respectively of 50% and 25%. (**f–g**) Final phenotypes (t = 6 min) corresponding to wild type with mesodermal radial intensity increased respectively of 50% and 25%.
(TIF)

Figure S8 **Meso-apical intensity study.** The quantitative effects of varying the intensity of mesodermal apical constriction in the time interval [−1.2 min, 2 min] (second invagination interval, Fig. 3c). (**a**) Meso-apical forces were increased/decreased by 25% and 50% of their value in the wild type case (Fig. 3c). (**b–c**) Different simulations refer to an increase/decrease of 25% and 50% in intensity. The perturbation of the intensity of apical constriction in the time interval does not substantially affect either the mesodermal/ectodermal thickness or the height of the furrow in the whole interval of invagination. (**d–e**) Final phenotypes (t = 6 min) corresponding to wild type with mesodermal apical intensity decreased respectively of 50% and 25%. (**f–g**) Final phenotypes (t = 6 min) corresponding to wild type with mesodermal apical intensity increased respectively of 50% and 25%.
(TIF)

Video V1 **Video of** ***in vivo*** **ventral furrow invagination: wild type genotype.** Video using multi-photon images of *in vivo* transverse cross-sections of a wild type embryo during ventral furrow formation. The embryo is labelled with Sqh-GFP and is oriented with its dorsal surface upward.
(AVI)

Video V2 **Video of** ***in silico*** **ventral furrow invagination: wild type genotype.** Video of computer simulation of *in silico* transverse cross-section of a wild type genotype. The embryo's 2D geometry is oriented with its dorsal side upward.
(AVI)

Video V3 **Video of** ***in vivo*** **ventral furrow invagination:** ***bnt*** **–mutant genotype.** Video using multi-photon images of *in vivo* transverse cross-sections of a *bnt-mutant* embryo during ventral furrow formation. The embryo is labelled with Sqh-GFP and is oriented with its dorsal surface upward.
(AVI)

Video V4 **Video of** ***in silico*** **ventral furrow invagination:** ***bnt-mutant*** **genotype.** Video of computer simulation of *in silico* transverse cross-section of a *bnt-mutant* genotype. The embryo's 2D geometry is oriented with its dorsal side upward.
(AVI)

Video V5 **Video of** ***in vivo*** **ventral furrow invagination:** ***arm-mutant*** **genotype.** Video using multi-photon images of *in vivo* transverse cross-sections of an *arm-mutant* embryo during ventral furrow formation. The embryo is labelled with Sqh-GFP and is oriented with its dorsal surface upward.
(AVI)

Video V6 **Video of** ***in silico*** **ventral furrow invagination:** ***arm-mutant*** **genotype.** Video of computer simulation of *in silico* transverse cross-section of an *arm-mutant* genotype. The embryo's 2D geometry is oriented with its dorsal side upward.
(AVI)

Video V7 **Video of** ***in vivo*** **ventral furrow invagination:** ***cta/t48*** **-mutant genotype.** Video using multi-photon images of *in vivo* transverse cross-sections of a *cta/t*48-mutant embryo during ventral furrow formation. The embryo is labelled with Sqh-GFP and is oriented with its dorsal surface upward.
(AVI)

Video V8 **Video of** ***in silico*** **ventral furrow invagination:** ***cta/t48*** **-mutant genotype.** Video of computer simulation of *in silico* transverse cross-section of a *cta/t48*-mutant genotype. The embryo's 2D geometry is oriented with its dorsal side upward.
(AVI)
