# Supplementary figures and images for: A Biomechanical Analysis of Ventral Furrow Formation in the Drosophila Melanogaster Embryo
Source: PLoS One. 2012 Apr 12;7(4):e34473. doi: 10.1371/journal.pone.0034473 (PMC3325263; doi:10.1371/journal.pone.0034473)

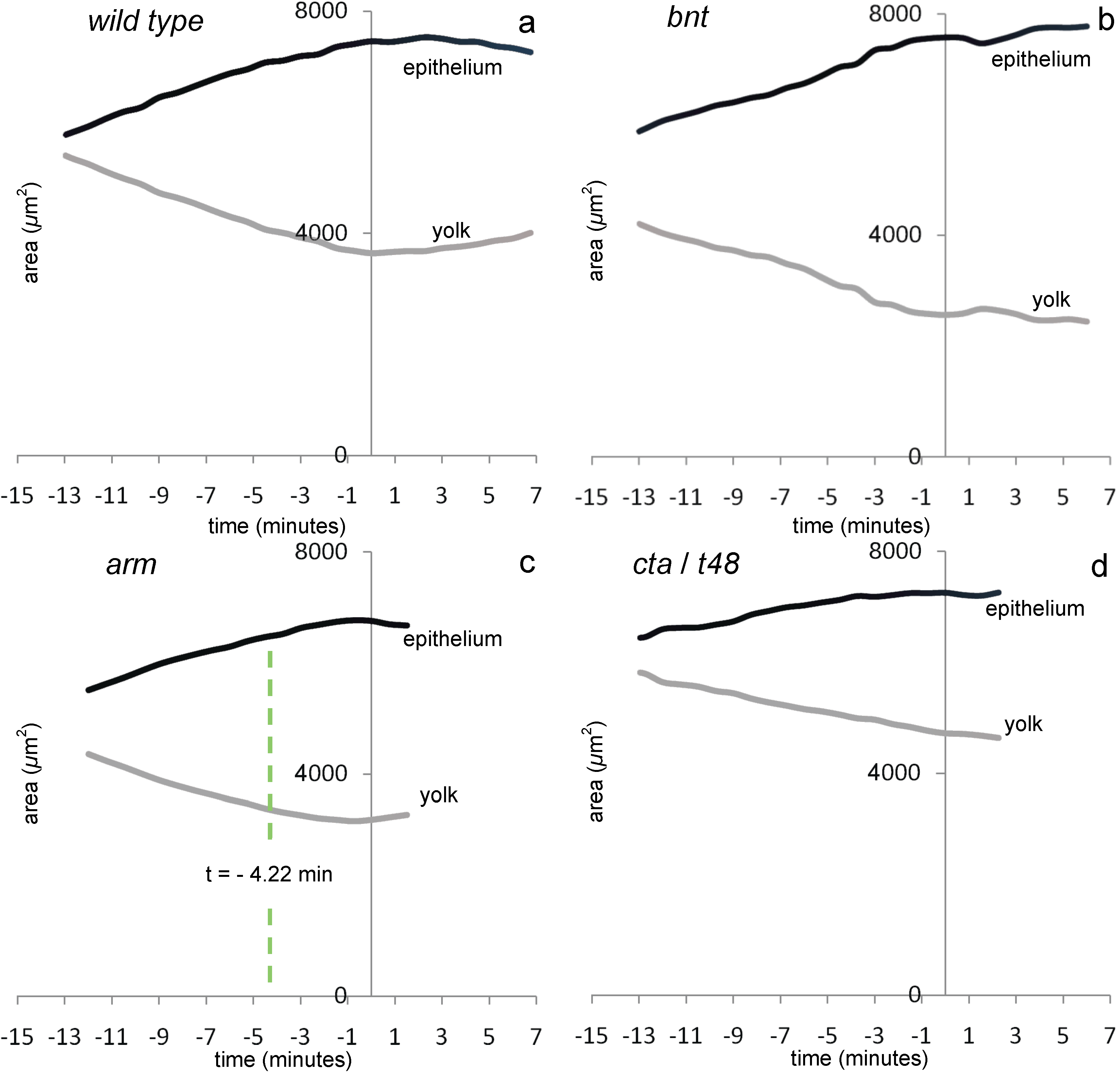

Supplement: Figure S1 — In vivo epithelium and yolk surface trends. Epithelium and yolk area trends versus time in different genotypes (one animal per genotype). Embryos were synchronized at t = 0 min using apical-basal cell height profiles on the dorsal side (Fig. 3). The average maximal extension of dorsal cells (t = 0 min) occurs at the maximal area of epithelium and minimal area of the yolk, which is a good indicator for the end of cellularisation of ectodermal cells. (c) cell-cell apical junctions in the arm mutant disrupt at t = −4.22 min (green vertical line), thus leading to the collapse of the ventrally denting furrow. (TIF) [file pone.0034473.s001.tif]

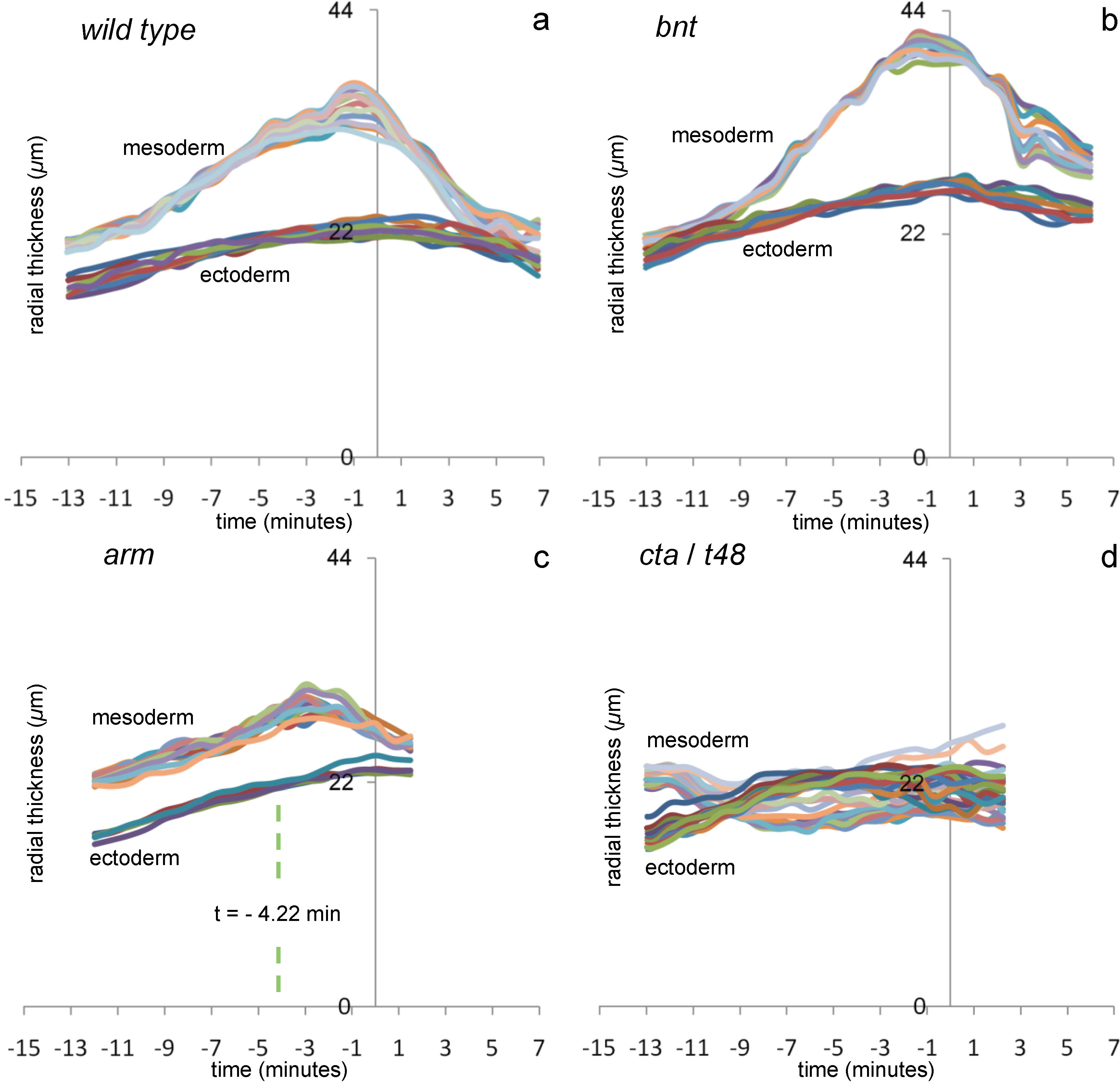

Supplement: Figure S2 — In vivo mesodermal and ectodermal radial thicknesses. Mesodermal and ectodermal trends versus time representing radial thicknesses across an angular span of tissue astride the ventral (V) and dorsal (D) points respectively. Measurements refer to a single animal per genotype. Mesodermal trends refer to radial thicknesses across an angular span of approximately 50 degrees astride ventral point V (25 degrees in each direction from V, figure 1a), whereas ectodermal trends refer to radial thicknesses across an angular span of approximately 120 degrees astride the dorsal point D (60 degrees in each direction from D, figure 1a. In vivo wt, bnt, arm and ct/t48 embryos were synchronized at t = 0 minutes by averaging the values of dorsal ectodermal cell length at each instant and aligning the maxima of such values for each genotype. (c) Cell-cell apical junctions in the arm mutant disrupt at t = −4.22 min (green vertical line), thus leading to the collapse of the ventrally denting furrow. (TIF) [file pone.0034473.s002.tif]

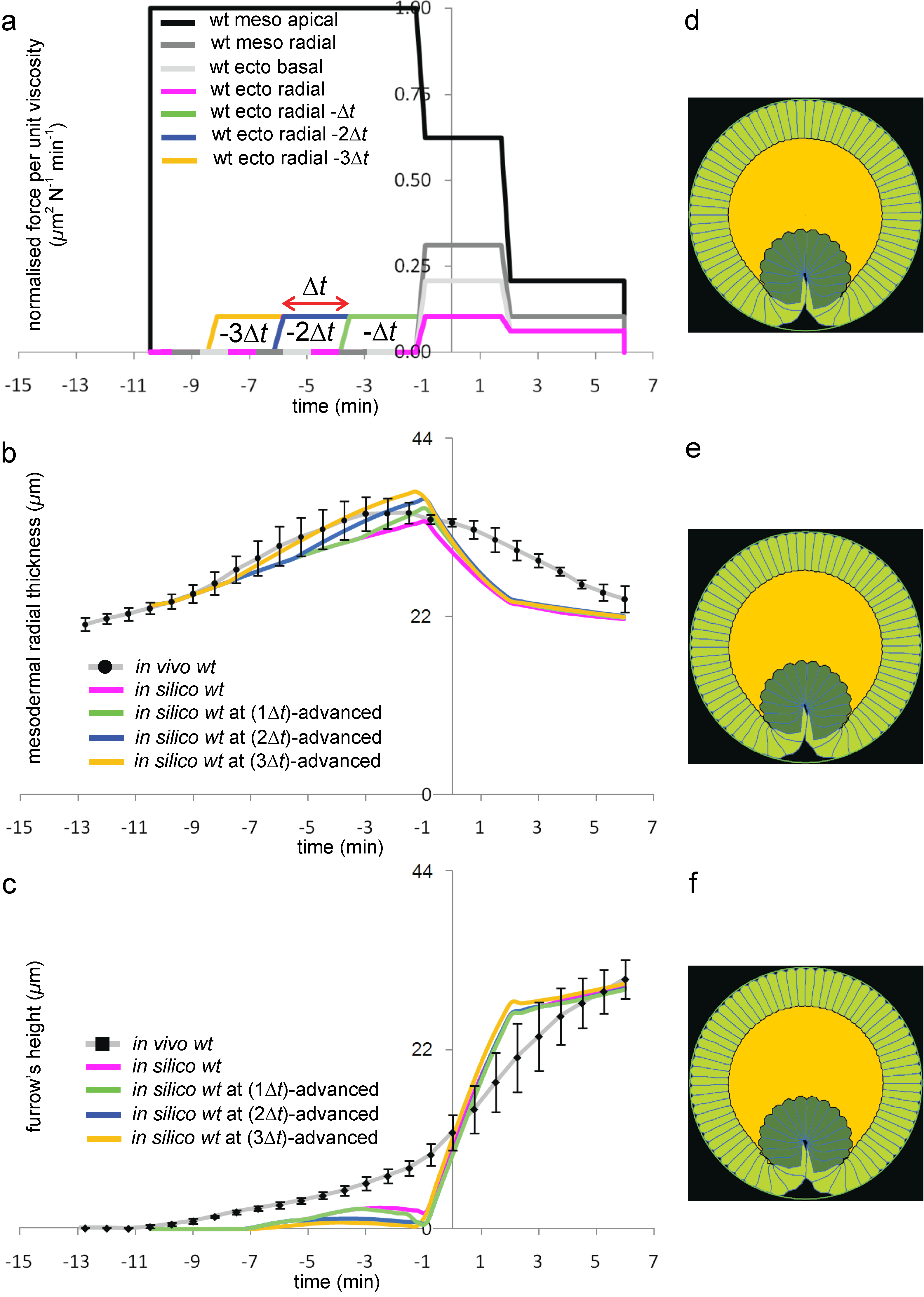

Supplement: Figure S3 — Ecto-radial time study. The quantitative effects of anticipating the onset of ectodermal radial shortening with respect to the wild type case reported in Fig. 3, while keeping the remaining force trends unchanged (Fig. 3c). (a) Force trend curves labelled by , and illustrate the case where ecto-radial movement was respectively advanced at t = −3.48 min, t = −5.8 min, t = −8.12 min with respect to the wt case (where ecto-radial movement onsets at t = −1.2 min, as shown in Fig. 3c). (b–c) changes in the onset instant of this movement with respect to the others has no significant effects on furrow's height h but impacts mesodermal rate of thickening, which increases with the anticipation of the movement. (d–f) Final phenotypes (t = 6 min) corresponding to wild type with ectodermal radial movements advanced respectively at , and . (TIF) [file pone.0034473.s003.tif]

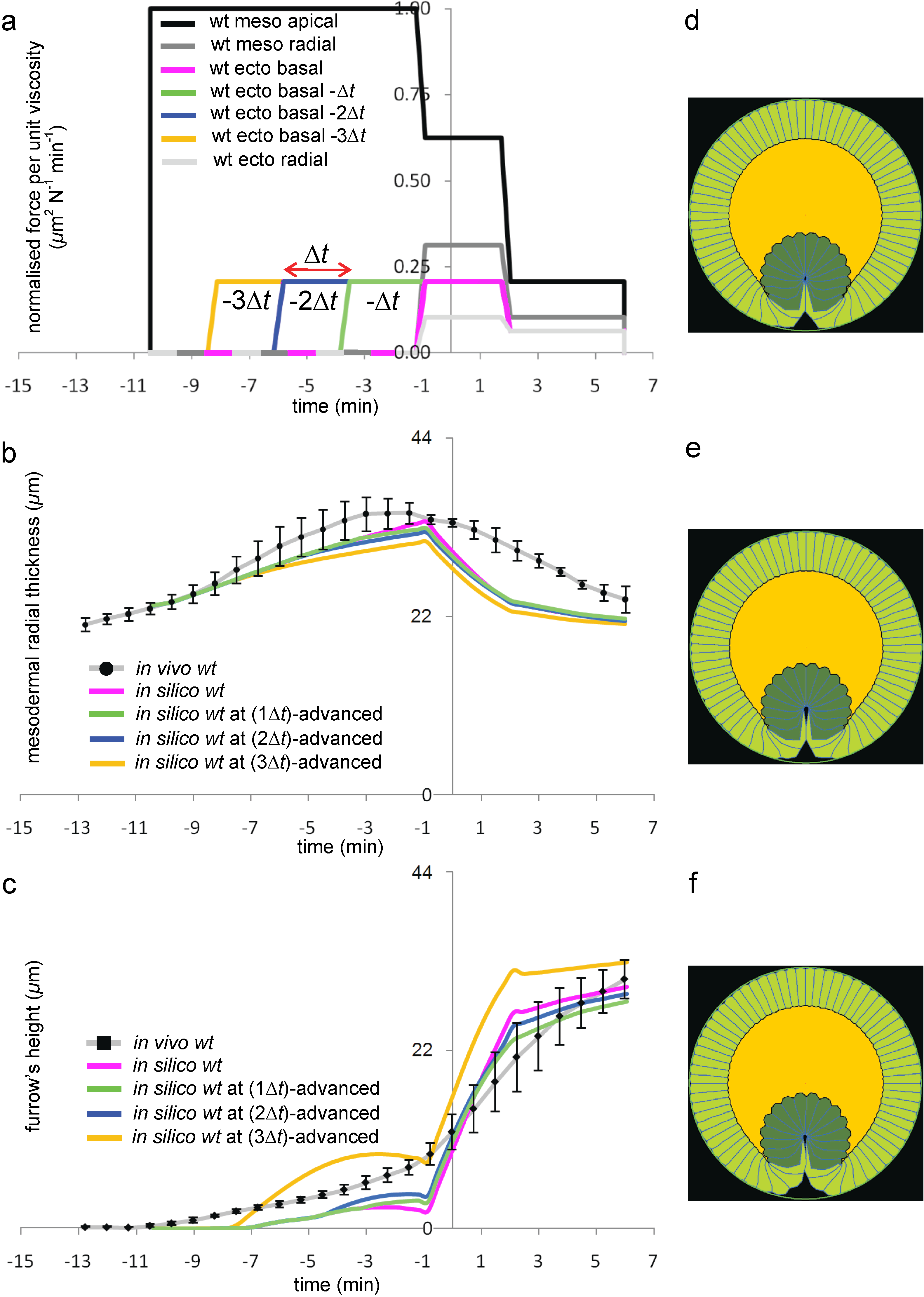

Supplement: Figure S4 — Ecto-basal time study. The quantitative effects of anticipating the onset of ectodermal basal constriction with respect to the wild type case reported in Fig. 3, while keeping the remaining force trends unchanged (Fig. 3c). (a) Force trend curves labelled by , and illustrate the case where ecto-basal movement was respectively advanced at t = −3.48 min, t = −5.8 min, t = −8.12 min with respect to the wt case (where ecto-basal movement onsets at t = −1.2 min, as shown in Fig. 3c). (b–c) changes in the onset time of this movement with respect to the others has significant effects on both mesodermal thickening ratio and furrow's height, which decrease with the anticipation of the movement (with the exception of yellow h trend due to numerical instabilities). (d–f) Final phenotypes (t = 6 min) corresponding to wild type with ectodermal basal movements advanced respectively at , and . (TIF) [file pone.0034473.s004.tif]

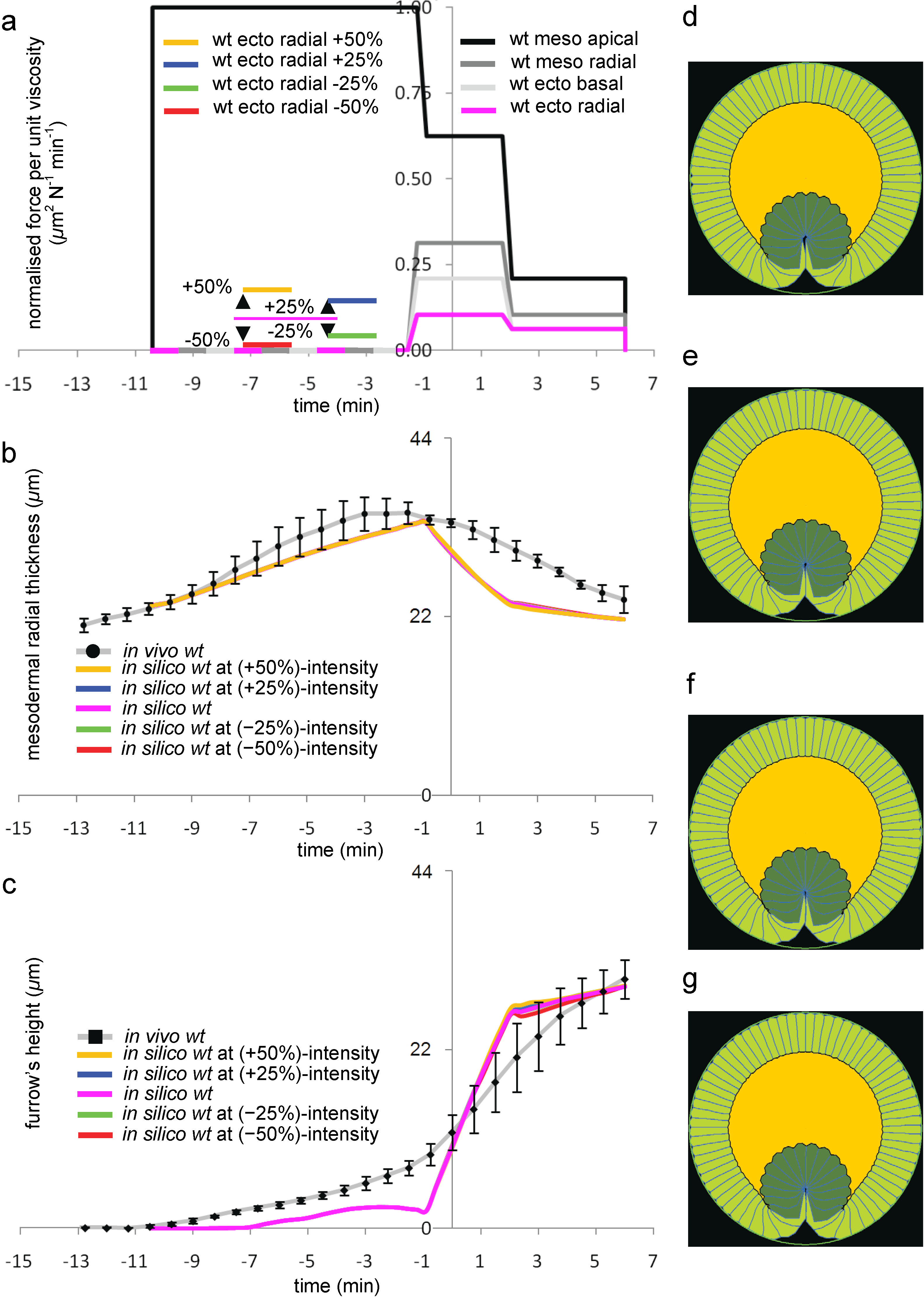

Supplement: Figure S5 — Ecto-radial intensity study. The quantitative effects of varying the intensity of ectodermal radial shortening in the time interval [−1.2 min,2 min] (second invagination interval, Fig. 3c). (a) Ecto-radial forces were increased/decreased by 25% and 50% of their value in the wild type case (Fig. 3c). (b–c) Different simulations refer to an increase/decrease of 25% and 50% in intensity. The perturbation of the intensity of apical constriction in the time interval does not substantially affect either the mesodermal/ectodermal thickness or the height of the furrow in the whole interval of invagination. (d–e) Final phenotypes (t = 6 min) corresponding to wild type with ectodermal radial intensity decreased respectively of 50% and 25%. (f–g) Final phenotypes (t = 6 min) corresponding to wild type with ectodermal radial intensity increased respectively of 50% and 25%. (TIF) [file pone.0034473.s005.tif]

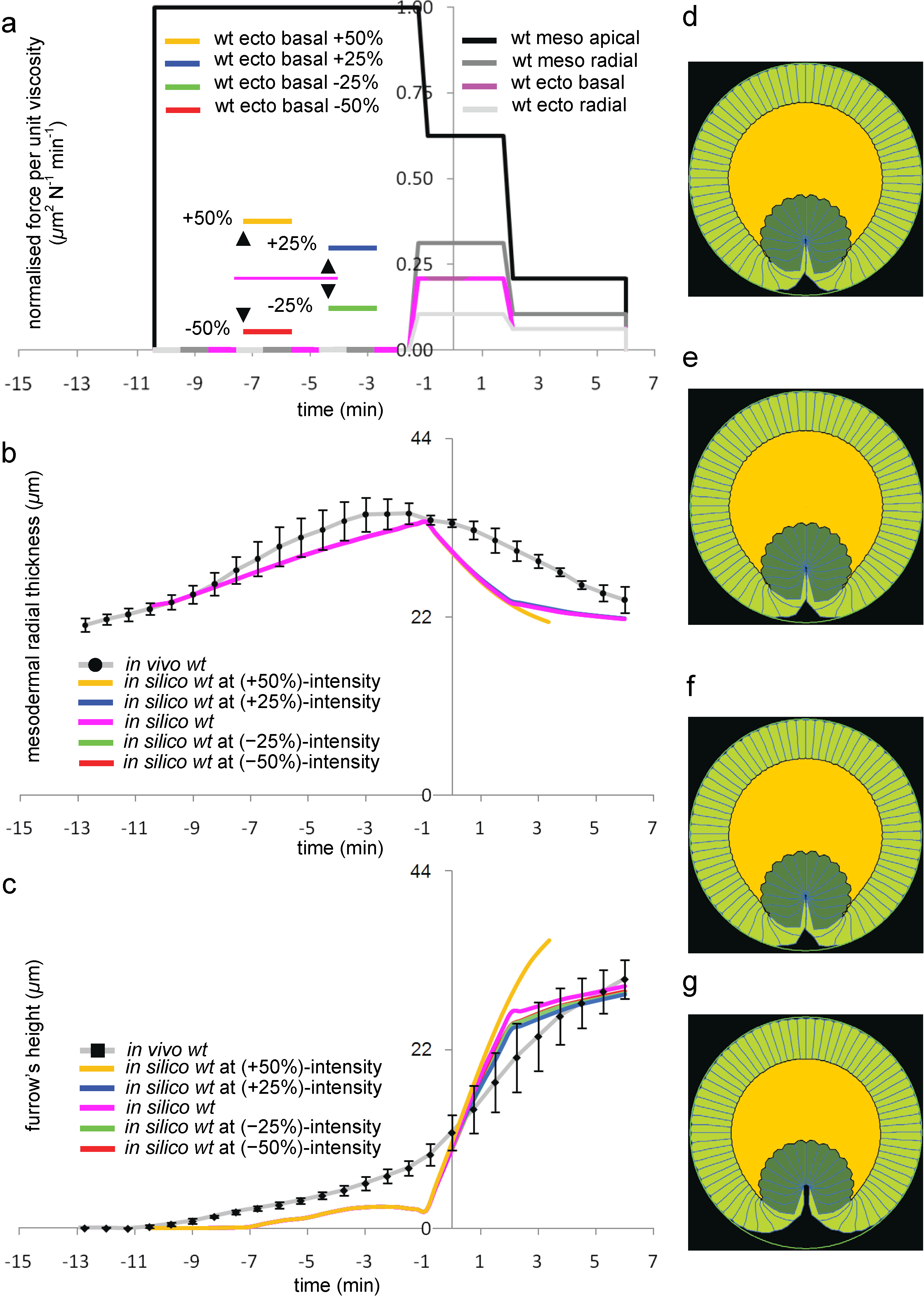

Supplement: Figure S6 — Ecto-basal intensity study. The quantitative effects of varying the intensity of ectodermal basal constriction in the time interval [−1.2 min,2 min] (second invagination interval, Fig. 3c). (a) Ecto-basal forces were increased/decreased by 25% and 50% of their value in the wild type case (Fig. 3c). (b–c) Different simulations refer to an increase/decrease of 25% and 50% in intensity. The perturbation of the intensity of basal constriction in the time interval does not substantially affect either the mesodermal/ectodermal thickness or the height of the furrow in the whole interval of invagination. (d–e) Final phenotypes (t = 6 min) corresponding to wild type with ectodermal basal intensity decreased respectively of 50% and 25%. (f–g) Final phenotypes (t = 6 min) corresponding to wild type with ectodermal basal intensity increased respectively of 50% and 25%. (TIF) [file pone.0034473.s006.tif]

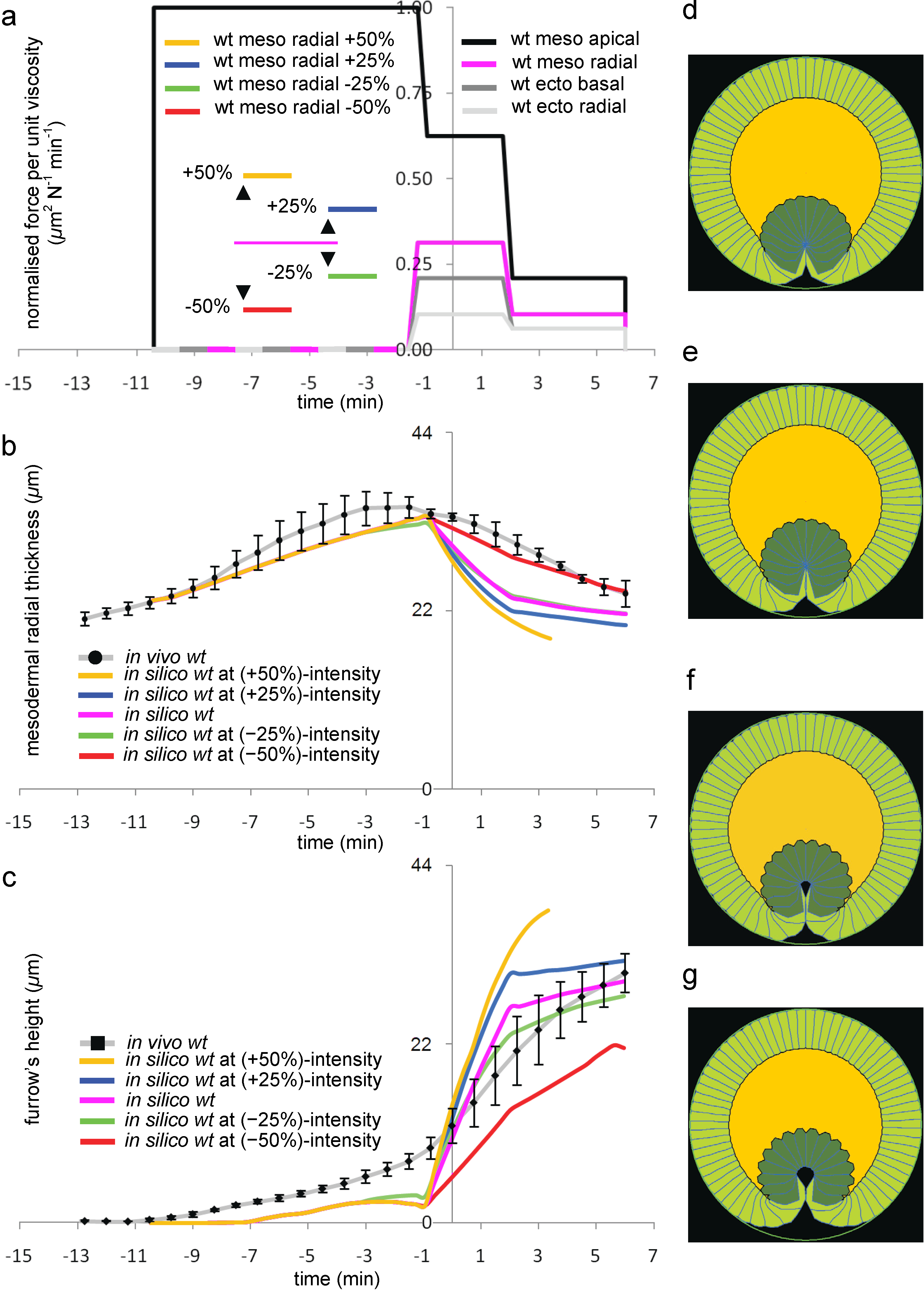

Supplement: Figure S7 — Meso-radial intensity study. The quantitative effects of varying the intensity of mesodermal radial shortening in the time interval [−1.2 min, 2 min] (second invagination interval, Fig. 3c). (a) Meso-radial forces were increased/decreased by 25% and 50% of their value in the wild type case (Fig. 3c). (b–c) Different simulations refer to an increase/decrease of 25% and 50% in intensity. The perturbation of the intensity of meso-radial forces in the time interval substantially affects both mesodermal thickness and height of the furrow in the whole interval of invagination. (d–e) Final phenotypes (t = 6 min) corresponding to wild type with mesodermal radial intensity decreased respectively of 50% and 25%. (f–g) Final phenotypes (t = 6 min) corresponding to wild type with mesodermal radial intensity increased respectively of 50% and 25%. (TIF) [file pone.0034473.s007.tif]

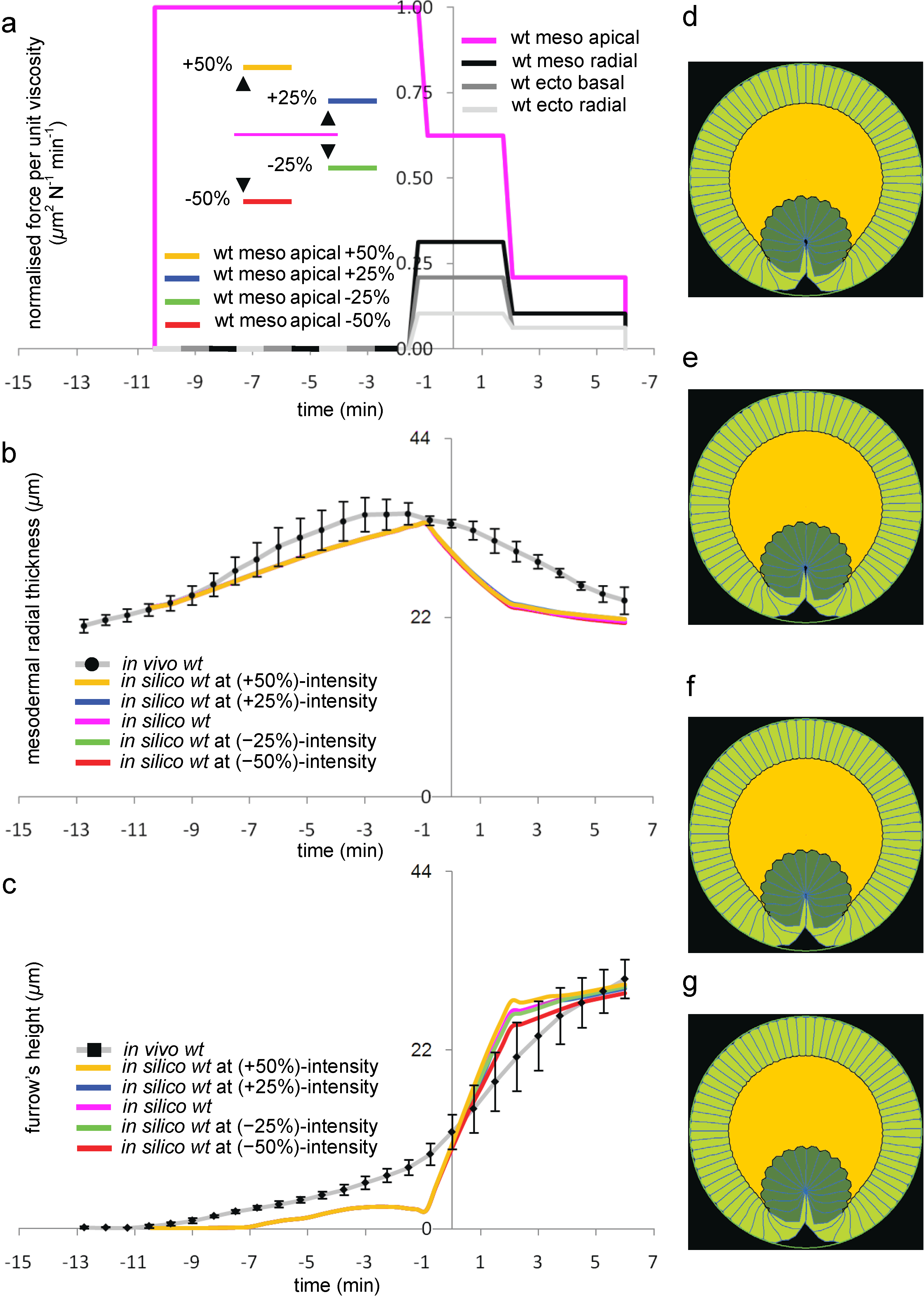

Supplement: Figure S8 — Meso-apical intensity study. The quantitative effects of varying the intensity of mesodermal apical constriction in the time interval [−1.2 min, 2 min] (second invagination interval, Fig. 3c). (a) Meso-apical forces were increased/decreased by 25% and 50% of their value in the wild type case (Fig. 3c). (b–c) Different simulations refer to an increase/decrease of 25% and 50% in intensity. The perturbation of the intensity of apical constriction in the time interval does not substantially affect either the mesodermal/ectodermal thickness or the height of the furrow in the whole interval of invagination. (d–e) Final phenotypes (t = 6 min) corresponding to wild type with mesodermal apical intensity decreased respectively of 50% and 25%. (f–g) Final phenotypes (t = 6 min) corresponding to wild type with mesodermal apical intensity increased respectively of 50% and 25%. (TIF) [file pone.0034473.s008.tif]
